# Cyclic carbamates from epoxides and isocyanates catalysed by inorganic salts

**DOI:** 10.1039/d5ra07585h

**Published:** 2025-12-19

**Authors:** Jordan Holland, Robbie A. Clark, Michael P. Shaver

**Affiliations:** a Department of Materials, University of Manchester Engineering Building A Oxford Road M13 9PL UK michael.shaver@manchester.ac.uk; b Sustainable Materials Innovation Hub, Henry Royce Institute, University of Manchester Manchester M13 9PL UK

## Abstract

Inexpensive inorganic salts can catalyse the insertion of isocyanates to epoxides without the addition of N-heterocyclic carbene cocatalysts. Optimising catalyst, solvent and reaction temperature offers high conversions to a range of cyclic carbamates with lower reaction temperatures and greener solvents. Catalyst recyclability is also enabled by switching to a polymeric PPNCl catalyst.

## Abstract 2

Simple lithium salts or recyclable polymeric cations catalyse the regioselective insertion of isocyanates into epoxides to afford oxazolidin-5-ones.

## Graphical abstract

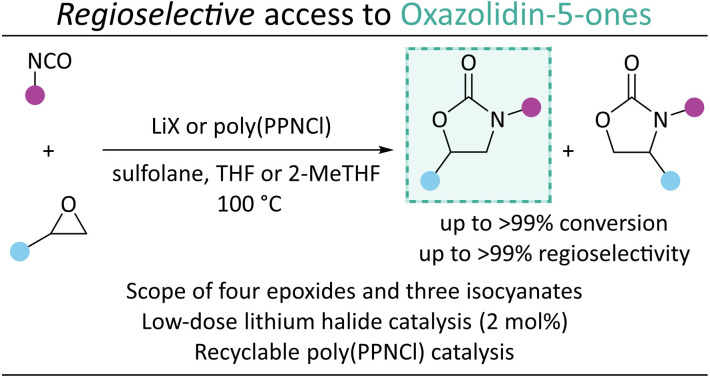

## Introduction

Cyclic carbamates are essential heterocyclic compounds growing in importance as small-molecule therapeutics including antibiotics, antidepressants, antivirals and agrichemicals in their pharmacophoric profile,^1,2^ broadening to solvents and chiral auxiliaries.^3,4^ Their synthesis is challenging, with toxic phosgene or CO_2_ insertion into aziridines at high temperatures and pressures,^5–8^ or isocyanate-epoxide coupling catalysed by complex ligand-supported metal catalysts,^9–12^ limiting reaction sustainability credentials. In addition, cyclic carbamate formation is hampered by the competitive formation of isocyanurate trimers. While these motifs are useful in thermoset resins, foams and aerogels, they are challenging to separate and limit reaction productivity when valuable cyclic products are the desired target.^13^

**Fig. 1:**
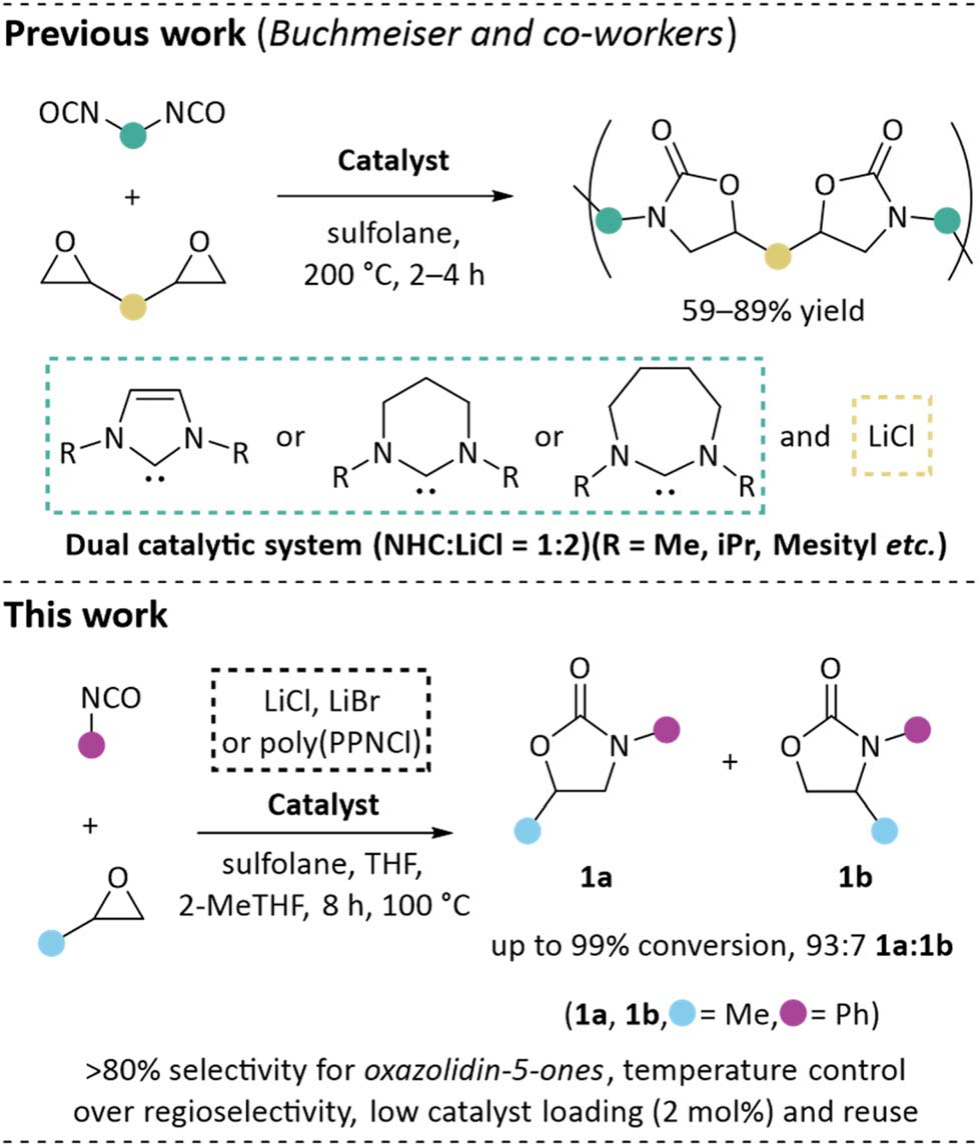
NHC/Lewis-acid catalysed production of poly(oxazolidine-5-ones) (top) and the use of simple salts and/or PPNCl to produce cyclic carbamates (bottom).

We have recently pioneered the development of polymeric frustrated Lewis pairs, poly(FLP)s, as recyclable catalysts for ring-opening and single-electron transfer reactions.^14–17^ With the development of N-heterocyclic carbene (NHC) variants of these systems,^18^ we were inspired by Buchmeiser and co-workers who showed linear poly(oxazolidin-2-one)s were formed from reactions of diepoxides and diisocyanates, catalysed by a cooperative catalyst system consisting of an NHC and metal halides such as LiCl in sulfolane (Fig. 1).^19^ When applying our poly(NHC) catalysts to the reaction of phenyl isocyanate with propylene oxide to form 1a, we noticed a reduction in conversion in the presence of NHCs. This contrasted with the Buchmeiser report that formed gels, characteristic of isocyanurate formation, in the absence of NHCs.^19^ Previous reports of simple inorganic salts catalysing the formation of specific bioactive targets from this coupling are rare.^20,21^ More complex salts, including squaramide/Bu_4_NI and tetraarlyphosphonium iodide binary catalysts,^22,23^ can also catalyse the process. Inspired by these previous efforts, we have explored both the use of simple salts, in absence of strong Lewis bases like NHCs, and complex salts designed for recyclability in the catalytic formation of cyclic carbamates.

## Results and discussion

We optimised our catalytic processes by coupling propylene oxide (PO) and phenylisocyanate (PhNCO) in sulfolane to form cyclic carbamates 1a and 1b at 120 °C as early trials showed that the reaction worked at this lower temperature and limited trimerization. Catalyst screening identified LiCl (75% conversion and selectivity for 1a to 1b of 87 : 13), LiBr (85%, 80 : 20) and bis(triphenylphosphine)iminium chloride, PPNCl (39%, 85 : 15), as promising catalysts (Table S1). In all cases, regioisomer 1a formed preferentially as nucleophilic attack of the anion at the less hindered carbon was favoured, with no isocyanurate side-products observed.

In recognition of the toxicity and environmental impacts of sulfolane, the effect of solvent on conversion and regioselectivity was explored (Table S2). Catalytic activity tracked with the ability of solvent to dissolve and stabilise the cations, as hypothesised previously for sulfolane.^24^ DMSO showed decreased conversions, suggesting coordination was too strong; chlorinated solvents poorly solubilise the lithium salts; no reaction was observed in toluene. We hypothesised that solvents of moderate polarity and weak Lewis basicity would afford ideal catalytic performance. Indeed, reactions in THF promoted quantitative conversion to the cyclic carbamate products with LiBr, while also improving regioselectivity (93 : 7). LiCl conversions were surprisingly low, however good conversion with PPNCl (82%, 89 : 11) was achieved. Importantly, we hoped to reduce dependence on fossil resources by testing 2-MeTHF, as it has shown promise as a greener solvent.^25^ While conversions were lower than for THF, with no reaction observed with LiCl, the promising result with LiBr (90%, 76 : 24) suggests optimisation of the reaction with green solvents may be possible in the future. Interestingly, PPNCl reactivity seemed dependent on solubility rather than innate reactivity. Based on these results, LiBr in THF was selected as the preferred catalyst system for further optimisation.

To further reduce the environmental burden of this reaction, we explored the impact of reaction temperature (Table S3). Increasing reaction temperatures to 140 °C maintained high conversions and avoided bis(phenyl)urea formation but showed decreased selectivity for 1a over 1b (79 : 21 *vs.* 93 : 7). Lower reaction temperatures decreased conversion (80 °C, 64%; 60 °C, 33%) and increased formation of the unwanted urea side product (Fig. S1). Temperature is thus highly important to control catalyst performance, as temperature can both mitigate side reactions and control regioselectivity, with 100 °C offering optimal performance.

**Fig. 2:**
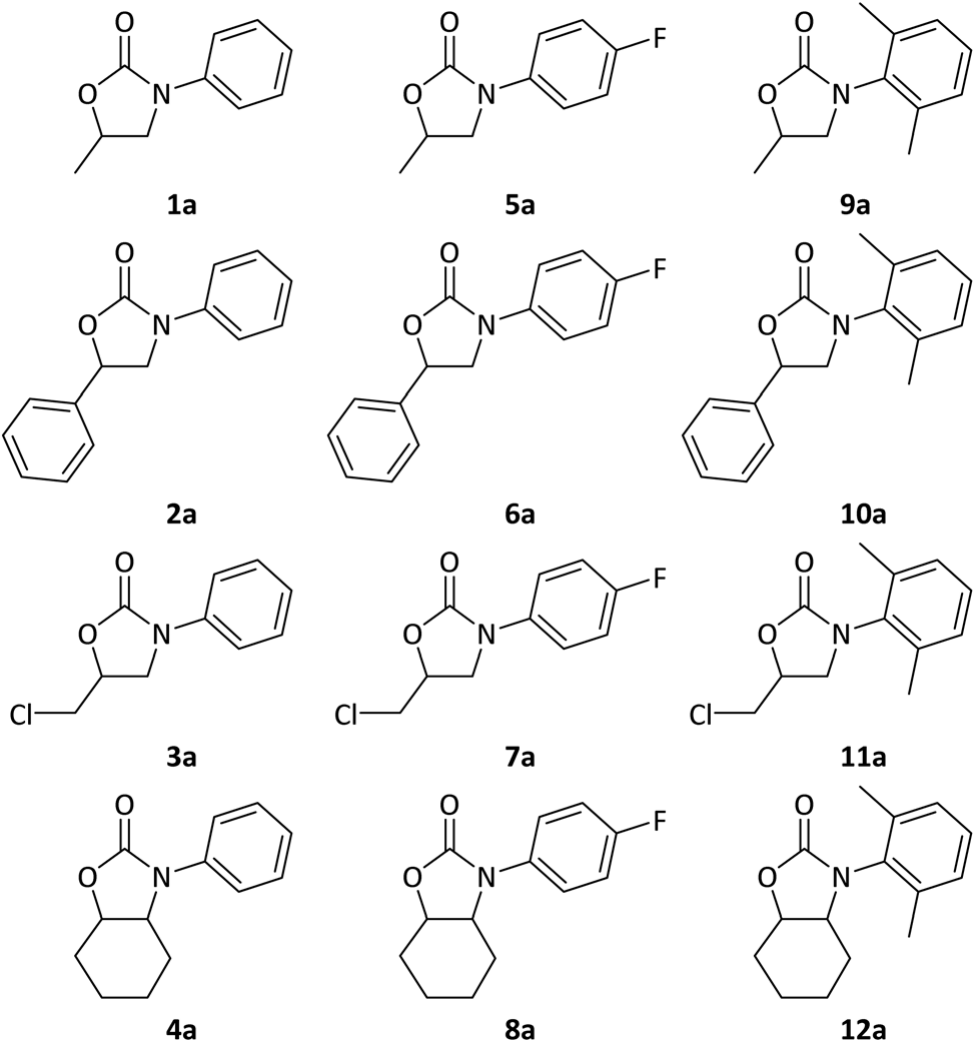
Cyclic carbamate scope. The oxazolidine-4-one regioisomer of each product is named as the b, as in Fig. 1.

With optimised conditions in hand, we sought to explore substrate scope (Fig. 2, and Table 1) using four epoxides (styrene oxide (SO), glycidyl chloride (GC), cyclohexene oxide (CHO) and the original PO) with three isocyanates (PhNCO, 4-fluorophenylisocyanate (FC_6_H_4_NCO), and 2,6-dimethylphenyl isocyanate (Me_2_C_6_H_3_NCO)). ^1^H-NMR spectroscopic analysis of the 12 resultant reactions was performed to determine conversions (Fig. S2–S13). ^13^C-NMR, ^19^F-NMR and Fourier-transform infrared (FTIR) characterisations are also given for 1a–12a, as applies, in the SI.

**Table 1:** Substrate scope for cyclic carbamate synthesis^a^

Product	Conversion^b^ (%)	*a* : *b* ratio^b^	CC : isocyanurate ratio^c^
1a	>99	100 : 0	100 : 0
2a	93	87 : 13	100 : 0
3a	>99	100 : 0	100 : 0
4a	15	—	100 : 0
5a	84	100 : 0	83 : 17
6a	82	85 : 15	82 : 18
7a	89	100 : 0	91 : 9
8a	5	—	4 : 96
9a	97	95 : 5	100 : 0
10a	94	87 : 13	100 : 0
11a	>99	100 : 0	100 : 0
12a	31	—	100 : 0

a THF, 100 °C, 8 h, LiBr (2 mol%).

b Conversions and *a* : *b* ratios determined by ^1^H-NMR spectroscopy of unpurified reaction mixture.

c Cyclic carbamate (CC) to isocyanurate ratio determined by ^1^H-NMR spectroscopy of residue after precipitation in water.

With PhNCO and Me_2_C_6_H_3_NCO, reactivity with PO, SO and GC was high, with 93–99% conversion, 0% isocyanurate formation, and up to 100% regioselectivity. The di-substituted CHO demonstrated lower conversions, attested to the steric bulk of the carbons inhibiting epoxide ring opening. Lower selectivities were observed for SO; while attack by the nucleophile occurs at the least-hindered carbon during insertion, the phenyl ring can stabilise the benzylic carbenium ion leading to more of the 3,4-isomer. When the electron-withdrawing FC_6_H_4_NCO was used as the isocyanate substrate, conversions were lower for all four epoxides (PO, 84%; SO, 82%; GC, 89%; CHO, 4.8%). More problematically, significant formation of isocyanurate was now observed, with 9–18% of the unwanted coupling product forming for the mono-substituted epoxides and isocyanurate trimer dominating in reactions with CHO (Fig. S14).

These results supported a pair of linked catalytic cycles (Fig. S15). Off-cycle trimerization occurs competitively by the isocyanate oxygen coordinated competitively to the Li^+^ prior to nucleophilic attack of the bromide anion to give the carbonyl-bromide intermediate. This nucleophilic nitrogen can react with two more isocyanates, followed by ring closure *via* bromide elimination to form the isocyanurate trimer. In most reactions, epoxide coordination to Li^+^ dominates, promoting ring opening by Br^−^ at the less bulky centre. Isocyanate insertion before ring closure gives the desired product. This highlights the importance of THF and 2-MeTHF as solvents, with their weak Lewis basicity offering control of the lithium coordination sphere. This can be controlled through judicious choice of reaction conditions, but is also highly substrate dependent, especially when substrate pairs are unreactive.

This substrate dependence also extended to non-aromatic isocyanates (Fig. S17). To explore the differential reactivity observed in the inspiring poly(oxazolidin-2-one) synthesis, we reacted the di-epoxide bisphenol A diglycidyl ether (BADGE) with hexamethylene diisocyanate (HDI) under our optimised conditions. Indeed, this alkyl isocyanate preferentially trimerises, forming cross-linked networks and precluding linear polymer formation, corroborating previous results.^19^

**Fig. 3:**
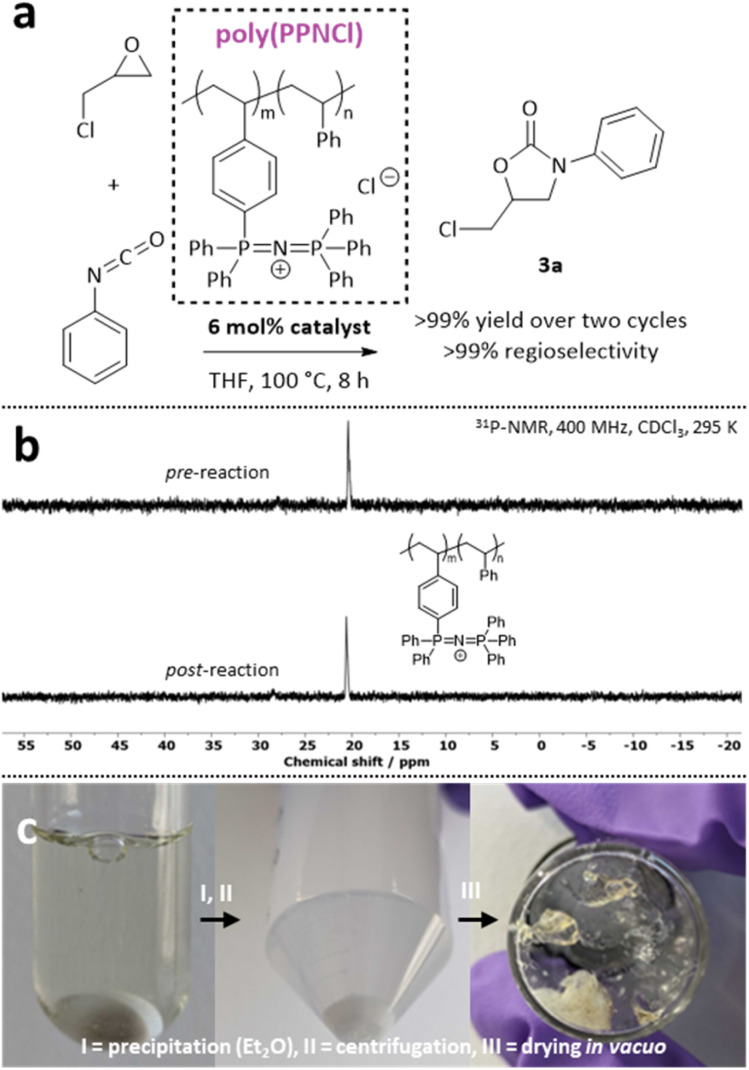
(a) Synthesis of 3a with poly(PPNCl) as catalyst. (b) ^31^P-NMR spectroscopy showcasing no catalyst oxidation after one cycle. (c) Recovery of the poly(PPNCl) catalyst.

While these simple salts offer improved reaction conditions, complex salts offer a complementary sustainability benefit. Surprised by the inefficiency of PPNCl in light of the previous report of phosphonium iodide catalysis,^23^ we revisit this bulky cation. PPNCl has garnered significant attention as a robust catalyst, particularly in epoxide insertion reactions.^26–29^ We suspected that observed conversion limitations were due to low catalyst solubility in the reaction media. We recently reported a polymeric version, poly(PPNCl) that is both recyclable and has vastly improved solubility.^29^ We showed that catalytic reactivity for a catalogue of reactions can be maintained over successive reaction cycles, with the catalyst being readily recovered by precipitation. With this in mind, we trialled poly(PPNCl) in an isocyanate insertion reaction to target a recyclable catalyst as a proof-of-concept (Fig. 3a). The system used was a styrenic copolymer with 6% loading of PPN^+^ units randomly distributed along the polymer chain. Upon coupling GC and PhNCO under our optimised reaction conditions, quantitative conversion of GC, with full regioselectivity and no isocyanurate formation was observed. Poly(PPNCl) was then recovered from the reaction mixture by simple precipitation. Followingly, the supernatant was decanted and the residue dried and reused (Fig. 3b and c). Catalytic performance was maintained in the successive reaction cycle (>99%), with no change to the regioselectivity of the obtained product or degradation of the polymeric catalyst by ^31^P-NMR spectroscopy. The poly(PPNCl) catalysed reaction may prevent trimerization due to the bulk of the cation, leaving only a main catalytic cycle similar to that of LiBr (Fig. S16).

This work serves as the foundation for future studies exploring catalyst recycling in flow reactor systems, with the recyclability essential to justify the higher environmental and economic cost of using complex salts.

## Conclusion

In conclusion, simple inorganic salts can catalyse the formation of cyclic carbamates, possibly offering improved sustainability. Both LiCl and LiBr readily catalyse the coupling of PO and PhNCO, with reaction optimisation giving best results when LiBr is used in THF (instead of sulfolane) at substantially lower temperatures (100 °C instead of 200 °C). The green solvent 2-MeTHF also facilitates the reaction but with lower conversions and selectivity. Twelve cyclic carbamates (1a–12a) were prepared, showing reaction versatility and helping understand competing mechanisms to avoid off-cycle isocyanate trimer formation. The designer cation PPNCl also catalyses the insertion reaction, however low solubility limits applicability. Use of the polymeric variant poly(PPNCl) improves solubility, activity and offers catalyst recyclability, further improving the process sustainability to make these important pharmacophoric compounds.

## Author contributions

Jordan Holland: conceptualisation, investigation, data curation, writing – review & editing. Robbie Clark: data curation, writing – review & editing. Michael Shaver: conceptualisation, writing – original draft, funding acquisition, supervision.

## Conflicts of interest

There are no conflicts of interest to declare.

## Supplementary Material

RA-015-D5RA07585H-s001

## Data Availability

The data supporting this article have been included as part of the supplementary information (SI) and include full experimental details, additional data and supporting figures and characterisation. Supplementary information: catalyst optimisation and spectroscopic characterisation of products. See DOI: https://doi.org/10.1039/d5ra07585h.

## References

[cit1] Prasher P., Mall T., Sharma M. (2023). Drug Dev. Res..

[cit2] Matošević A., Bosak A. (2020). Arch. Ind. Hyg. Toxicol..

[cit3] Gzara L., Chagnes A., Carré B., Dhahbi M., Lemordant D. (2006). J. Power Sources.

[cit4] Davies S. G., Fletcher A. M., Robers P. M., Thomson J. E. (2019). Org. Biomol. Chem..

[cit5] Pulla S., Felton C. M., Ramidi P., Gartia Y., Ali N., Nasini U. B., Ghosh A. (2013). J. CO_2_ Util..

[cit6] Zhang Z., Ye J.-H., Wu D.-S., Zhou Y. Q., Yu D.-G. (2018). Chem.–Asian J..

[cit7] Arshadi S., Banaei A., Ebrahimiasl S., Monfared A., Vessally E. (2019). RSC Adv..

[cit8] Miller A. W., Nguyen S. T. (2004). Org. Lett..

[cit9] Baronsky T., Beattie C., Harrington R. W., Irfan R., North M., Osende J. G., Young C. (2013). ACS Catal..

[cit10] Paddock R. L., Adhikari D., Lord R. L., Baik M.-H., Nguyen S. T. (2014). Chem. Commun..

[cit11] Beattie C., North M. (2014). RSC Adv..

[cit12] Barros M. T., Phillips A. M. F. (2010). Tetrahedron:Asymmetry.

[cit13] Wu X., Mason J., North M. (2017). Chem.–Eur. J..

[cit14] Wang M., Shanmugam M., McInnes E., Shaver M. P. (2023). J. Am. Chem. Soc..

[cit15] Horton T. A. R., Wang M., Shaver M. P. (2022). Chem. Sci..

[cit16] Yolsal U., Wang M., Royer J. R., Shaver M. P. (2019). Macromolecules.

[cit17] Wang M., Nudelman F., Matthes R., Shaver M. P. (2017). J. Am. Chem. Soc..

[cit18] Holland J., Shaver M. P. (2025). Can. J. Chem..

[cit19] Altmann H. J., Clauss M., König S., Frick-Delaittre E., Koopmans C., Wolf A., Guertler C., Naumann S., Buchmeiser M. R. (2019). Macromolecules.

[cit20] Ashida K., Frisch K. C. (1972). J. Cell. Plast..

[cit21] Ghrab S., Aroua L., Beji M. (2017). J. Heterocycl. Chem..

[cit22] Rostami A., Ebrahimi A., Husband J., Anwar M. U., Csuk R., Al-Harrasi A. (2020). Eur. J. Org Chem..

[cit23] Toda Y., Gomyou S., Tanaka S., Komiyama Y., Kikuchi A., Suga H. (2017). Org. Lett..

[cit24] Xuan X., Wang J., Lu J., Pei N., Mo Y. (2001). Spectrochim. Acta, Part A.

[cit25] Englezou G., Kortsen K., Pacheco A. A., Cavanagh R., Lentz J. C., Krumins E., Sanders-Velez C., Howdle S. M., Nedoma A. J., Taresco V. (2020). J. Polym. Sci..

[cit26] Buonerba A., De Nisi A., Grassi A., Milione S., Capacchione C., Vagin S., Rieger B. (2015). Catal. Sci. Technol..

[cit27] Sibaouih A., Ryan P., Leskelä M., Rieger B., Repo T. (2009). Appl. Catal., A.

[cit28] Chen F., Tao S., Liu N., Guo C., Dai B. (2021). Appl. Organomet. Chem..

[cit29] Xu Z., Wang M., Shaver M. P. (2024). Chem. Sci..

