# Supplementary material for: Cyclic carbamates from epoxides and isocyanates catalysed by inorganic salts
Source: RSC Adv. 2025 Dec 19;15(59):51120–3. doi: 10.1039/d5ra07585h (PMC12716260; doi:10.1039/d5ra07585h)
Supplement: RA-015-D5RA07585H-s001 [file RA-015-D5RA07585H-s001.pdf]

Supplementary Information (SI) for RSC Advances.
This journal is © The Royal Society of Chemistry 2025

Cyclic Carbamates from Epoxides and Isocyanates Catalysed by Inorganic Salts

Supplementary Information

Jordan Holland[a,b], Robbie A. Clark[a,b] and Michael P. Shaver[a,b]*

[a] Department of Materials, Engineering Building A, University of Manchester, Oxford Road, M13 9PL, U.K.

[b] Sustainable Materials Innovation Hub, Henry Royce Institute, University of Manchester, Manchester, M13 9PL, U.K.

michael.shaver@manchester.ac.uk

Contents

Table S1 Catalyst screen for propylene oxide and phenyl isocyanate coupling

Table S2 Solvent screen for propylene oxide and phenyl isocyanate coupling

Table S3 Temperature screen for propylene oxide and phenyl isocyanate coupling with LiBr

Fig. S1 ^{1}H-NMR spectra of crude material aromatic region from PhNCO/PO couplings at 60 °C (a), 80 °C (b), and 100 °C (c)

Fig. S2 ^{1}H-NMR spectrum of products **1a/1b** from PO/PhNCO coupling in THF with LiBr

Fig. S3 ^{1}H-NMR spectrum of products **2a/2b** from SO/PhNCO coupling in THF with LiBr

Fig. S4 ^{1}H-NMR spectrum of products **3a/3b** from GC/PhNCO coupling in THF with LiBr

Fig. S5 ^{1}H-NMR spectrum of products **4a/4b** from CHO/PhNCO coupling in THF with LiBr

Fig. S6 ^{1}H-NMR spectrum of products **5a/5b** from PO/FC_6H_4NCO coupling in THF with LiBr

Fig. S7 ^{1}H-NMR spectrum of products **6a/6b** from SO/FC_6H_4NCO coupling in THF with LiBr

Fig. S8 ^{1}H-NMR spectrum of products **7a/7b** from GC/FC_6H_4NCO coupling in THF with LiBr

Fig. S9 ^{1}H-NMR spectrum of products **8a/8b** from CHO/FC_6H_4NCO coupling in THF with LiBr

Fig. S10 ^{1}H-NMR spectrum of products **9a/9b** from PO/$Me_2C_6H_3NCO$ coupling in THF with LiBr

Fig. S11 ^{1}H-NMR spectrum of products **10a/10b** from SO/$Me_2C_6H_3NCO$ coupling in THF with LiBr

Fig. S12 ^{1}H-NMR spectrum of products **11a/11b** from GC/$Me_2C_6H_3NCO$ coupling in THF with LiBr

Fig. S13 ^{1}H-NMR spectrum of products **12a/12b** from CHO/$Me_2C_6H_3NCO$ coupling in THF with LiBr

Fig. S14 ^{13}C-NMR spectrum of 5-methyl-3-phenyloxazolidin-2-one (**1a**) in THF-d_8

Fig. S15 ^{13}C-NMR spectrum of 3,5-diphenyloxazolidin-2-one (**2a**) in THF-d_8

Fig. S16 ^{13}C-NMR spectrum of 5-(chloromethyl)-3-phenyloxazolidin-2-one (**3a**) in THF-d_8

Fig. S17 ^{13}C-NMR spectrum of 4,5-tetramethylene-3-phenyl-1,3-oxazolidin-2-one (**4a**) in THF-d_8

Fig. S18 ^{13}C-NMR spectrum of 3-(4-fluorophenyl)-5-methyloxazolidin-2-one (**5a**) in THF-d_8

Fig. S19 ^{13}C-NMR spectrum of 3-(4-fluorophenyl)-5-phenyloxazolidin-2-one (**6a**) in THF-d_8

Fig. S20 ^{13}C-NMR spectrum of 3-(4-fluorophenyl)-5-chloromethyloxazolidin-2-one (**7a**) in THF-d_8

Fig. S21 ^{13}C-NMR spectrum of 3-(4-fluorophenyl)-hexahydrobenzooxazol-2-one (**8a**) in THF-d_8

Fig. S22 ^{13}C-NMR spectrum of 3-(2,6-dimethylphenyl)-5-methyloxazolidin-2-one (**9a**) in THF-d_8

Fig. S23 ^{13}C-NMR spectrum of 3-(2,6-dimethylphenyl)-5-phenyloxazolidin-2-one (1**0a**) in THF-d_8

Fig. S24 ^{13}C-NMR spectrum of 5-(chloromethyl)-3-(2,6-dimethylphenyl)oxazolidin-2-one (**11a**) in THF-d_8

Fig. S25 ^{13}C-NMR spectrum of 3-(2,6-dimethylphenyl)hexahydrobenzo[d]oxazol-2(3H)-one (**12a**) in THF-d_8

Fig. S26 (a) Isocyanurate side product generated from 1-fluoro-4-isocyanatobenzene (FC_6H_4NCO) with either propylene oxide (PO), styrene oxide (SO), glycidyl chloride (GC) or cyclohexene oxide (CHO). (b) ^{1}H-NMR spectrum of the aromatic region of FC_6H_4NCO from **5a** formation (c) ^{1}H-NMR spectrum of post-precipitation crude product from 5a formation. (d) ^{19}F-NMR spectrum of post-precipitation crude product from 5a formation

Fig. S27 Proposed catalytic cycles for the formation of cyclic carbamates and isocyanurates meditated by LiBr

Fig. S28 Proposed catalytic cycle for the formation of cyclic carbamates and isocyanurates meditated by poly(PPNCl)

Fig. S29 Reaction of BADGE with HDI leading to formation of a cross-linked isocyanurate

References

Experimental

General Considerations

All reactions and purifications requiring inert conditions, were performed under an argon or nitrogen atmosphere, unless stated. Air-sensitive reactions were performed using either a dual-manifold Schlenk line equipped with an in-line gas drying column containing copper catalysts and molecular sieves, or in an argon/nitrogen-filled glovebox. All reagents and solvents used in air and moisture sensitive reactions were dried and degassed prior to use. Glassware used in air and moisture sensitive reactions was pre-dried in an oven (200 °C) for a minimum of 12 h.

Materials

All commercially available reagents were purchased from Acros Organics, AlfaAesar, Fluorochem, Fisher Scientific, Sigma Aldrich or TCI and used as received unless otherwise stated. Anhydrous $CHCl_3$, DCM, n-hexanes, THF and toluene was obtained from an MBraun 7 Solvent Purification System containing alumina and copper catalysts and degassed by multiple freeze-pump-thaw cycles. Dry DMSO and sulfolane were prepared by stirring over calcium hydride (CaH_2, Sigma Aldrich, 99 %) overnight before distillation under reduced pressure. Both solvents were then stored over 3 Å molecular sieves for 7 days prior to use. Dry methanol was acquired by being flushed with N_2 overnight before being placed over 3 Å molecular sieves for 5 days before use. All deuterated solvents were stirred over CaH_2 and distilled under inert conditions. All cyclic ether substrates were stirred overnight and distilled before being stored at – 35 °C in the glovebox freezer. Isocyanates were degassed by flushing with N_2 for 2 h prior to being stored over 3 Å molecular sieves at 2–4 °C. Styrene was dried over CaH_2 overnight and distilled under vacuum before being stored in the glovebox freezer at – 35 °C. Poly(PPNCl) was synthesised by Dr. Meng Wang and Ziwei Xu respectively, the synthetic route of which can be found in our recent publications.[1,2] Magnesium turnings were dried at 200 °C for 3 days prior to their application in Grignard reactions. Carbon dioxide (N5.0, research grade, 99.999% purity) and hydrogen (research grade, 99.99 % purity) were purchased from BOC.

Instrumentation

All nuclear magnetic resonance (NMR) spectroscopy data was obtained using a 400 MHz Bruker AVII (BBFO 5 mm probe), 400 MHz Bruker AVIII HD (BBO 5 mm probe) or 500 MHz Bruker AVIII HD (BBO 5 mm probe) NMR spectrometer. All air/moisture sensitive samples were analysed in J Young's tap NMR tubes. Centrifugation of fine polymer precipitates was completed using an Eppendorf Benchtop Centrifuge 5810.

General Procedure for Cyclic Carbamate Preparation

The stated procedure is based on phenylisocyanate insertion to propylene oxide. This procedure was amended based on choice of catalyst, solvent, temperature, duration, epoxide or isocyanate. Within a glovebox, LiBr (3.3 mg, 36 μmol) and 1,3,5-trimethoxybenzene (TMB) were dissolved in THF (3.0 mL) and transferred to an ampoule. A solution of propylene oxide (0.11 g, 1.8 mmol) in THF (2.8 mL) was added to the reaction vessel and removed from the glovebox. The ampoule was then cycled onto a Schlenk line before phenyl isocyanate (0.2 mL, 1.8 mmol) was added, whilst stirring vigorously. A small amount of the reaction mixture was extracted for analysis by 1H NMR spectroscopy. The reaction was then heated at 100 °C for 8 h. After completion, the reaction was quenched by cooling in ice and analysed by 1H NMR spectroscopy

to calculate conversions. Starting materials were removed by precipitation into cold deionised water, and the precipitate isolated by filtration before drying the obtained solid under vacuum.

Table S1 Catalyst screen for propylene oxide and phenyl isocyanate (**PhNCO**) coupling.

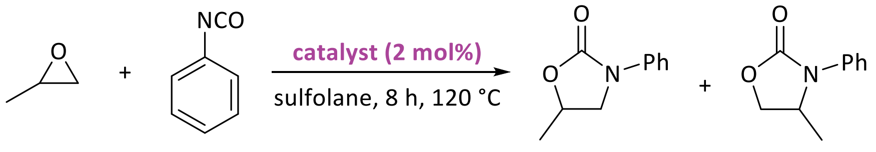

Entry	**PhNCO** Equiv.	Catalyst	Conversion (%)[a]	1a:1b[a]
1	1	-	NR	-
2	-	LiCl	NR	-
3	1	LiCl	75	87:13
4	1	LiF	NR	-
5	1	LiBr	85	80:20
6	1	LiO*t*Bu	NR	-
7	1	NaCl	NR	-
8	1	PPNCl	39	85:15
9	1	$ZnCl_2$	NR	-

[a]determined by ^{1}H-NMR spectroscopy. NR = no reaction.

Table S2 Solvent screen for propylene oxide and phenyl isocyanate (**PhNCO**) coupling.

Entry	Catalyst	Solvent	Conversion (%)[a]	1a:1b[a]
1	LiCl	DMSO	29	81:19
2	LiBr	DMSO	22	63:37
3	PPNCl	DMSO	18	83:17
4	LiCl	$CHCl_3$	NR	-
5	LiBr	$CHCl_3$	NR	-
6	PPNCl	$CHCl_3$	62	82:18
7	LiCl	DCM	NR	-
8	LiBr	DCM	NR	-
9	PPNCl	DCM	53	81:19
10	LiCl	Toluene	NR	-
11	LiBr	Toluene	NR	-
12	PPNCl	Toluene	NR	-
13	LiCl	THF	34	88:12
14	LiBr	THF	>99	93:7
15	PPNCl	THF	82	89:11
16	LiCl	2-MeTHF	NR	-
17	LiBr	2-MeTHF	90	76:24
18	PPNCl	2-MeTHF	64	80:20

[a]determined by ^{1}H-NMR spectroscopy. NR = no reaction.

Table S3 Temperature screen for propylene oxide and phenyl isocyanate (**PhNCO**) coupling with LiBr.

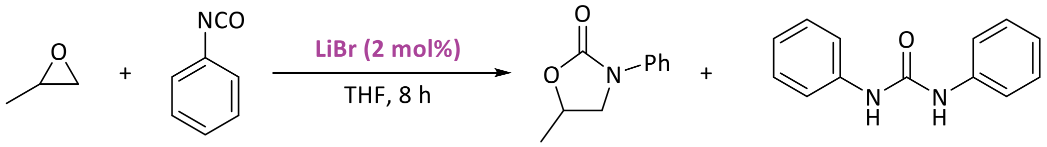

1a bis(phenyl)urea

Entry	T (°C)	Conversion (%)[a]	1a:1b[a]	1a:urea[a]
1	60	33	93:7	18:82
2	80	64	93:7	55:45
3	100	>99	93:7	100:0
4	140	>99	79:21	100:0

[a]determined by ^{1}H-NMR spectroscopy.

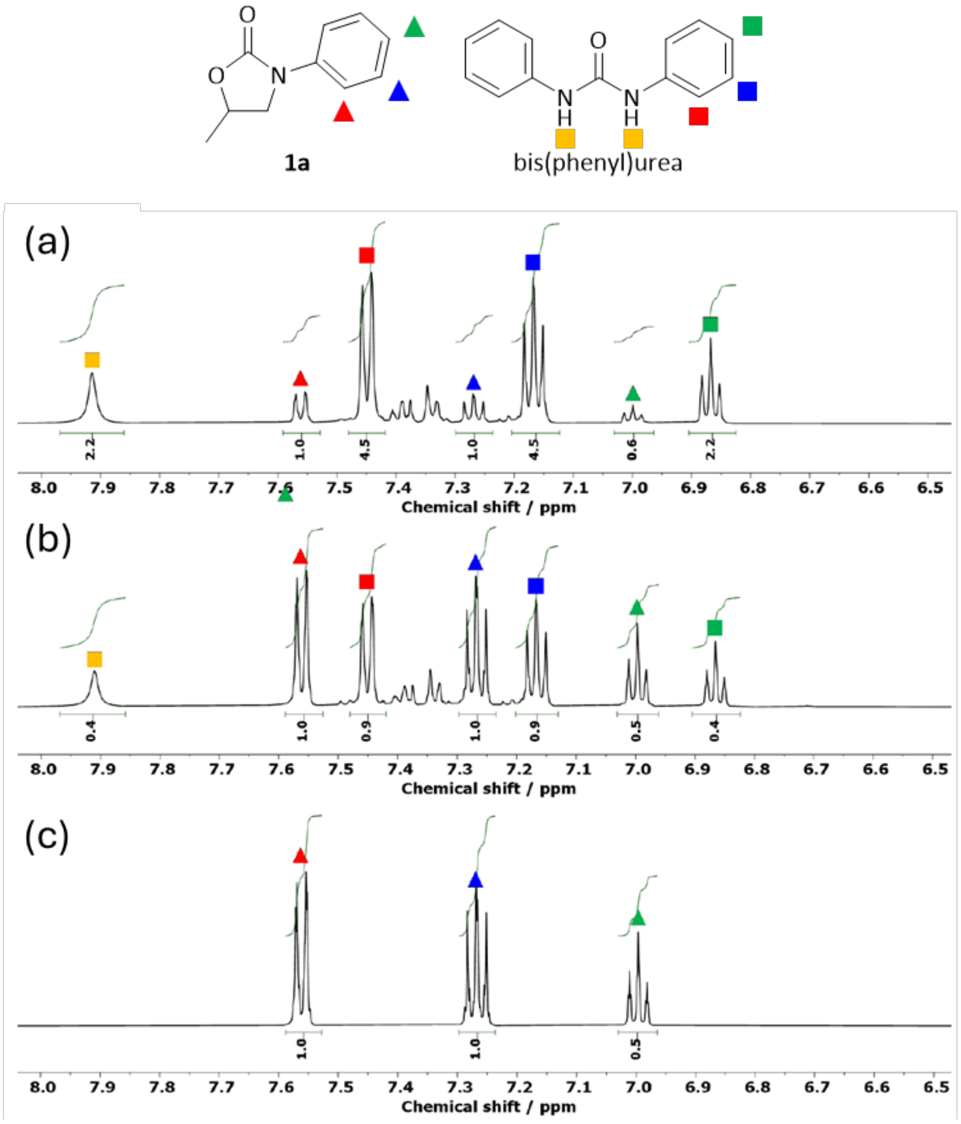

Fig. S1 ^{1}H-NMR spectra (400 MHz, $CDCl_3$, 295 K) of crude material aromatic region from PhNCO/PO couplings (**Table S3**) at 60 °C (a), 80 °C (b), and 100 °C (c).

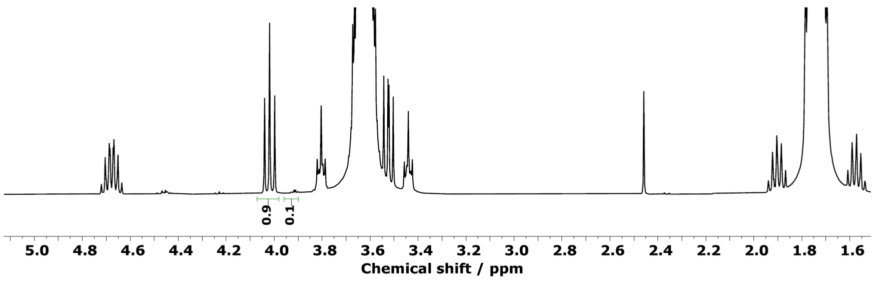

Fig. S2 Product/reagent region of ^{1}H-NMR spectrum of products **1a**/**1b** from PO/PhNCO coupling in THF with LiBr.

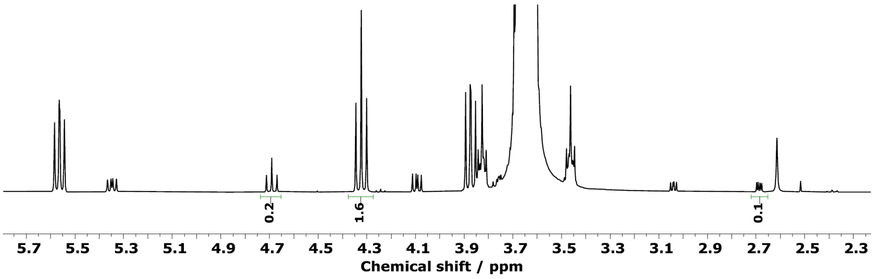

Fig. S3 Product/reagent region of ^{1}H-NMR spectrum of products **2a**/**2b** from SO/PhNCO coupling in THF with LiBr.

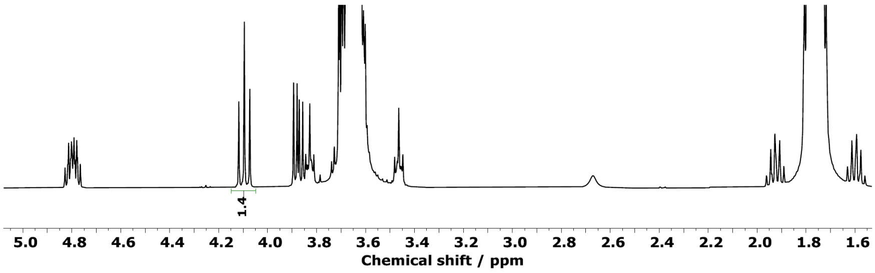

Fig. S4 Product/reagent region of ^{1}H-NMR spectrum of products **3a**/**3b** from GC/PhNCO coupling in THF with LiBr.

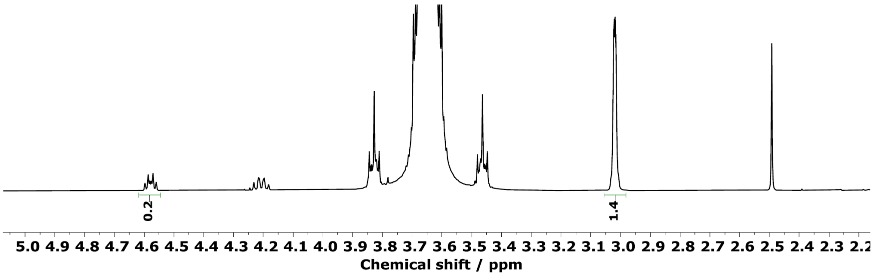

Fig. S5 Product/reagent region of ^{1}H-NMR spectrum of products **4a**/**4b** from CHO/PhNCO coupling in THF with LiBr.

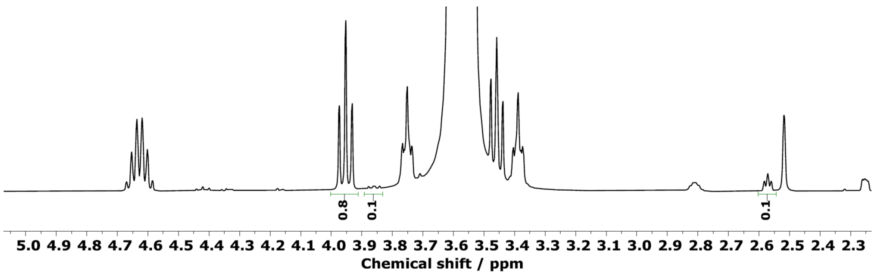

Fig. S6 Product/reagent region of ^{1}H-NMR spectrum of products **5a**/**5b** from PO/ FC_6H_4NCO coupling in THF with LiBr.

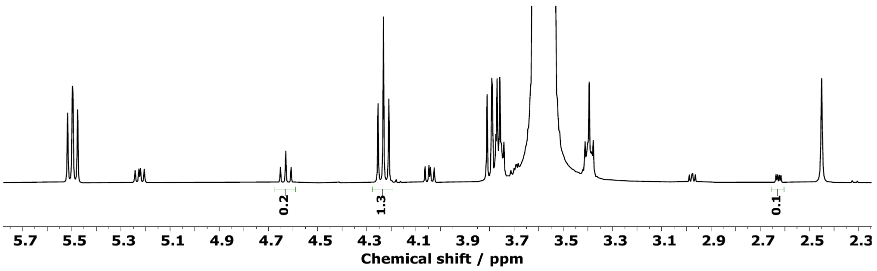

Fig. S7 Product/reagent region of ^{1}H-NMR spectrum of products **6a**/**6b** from SO/ FC_6H_4NCO coupling in THF with LiBr.

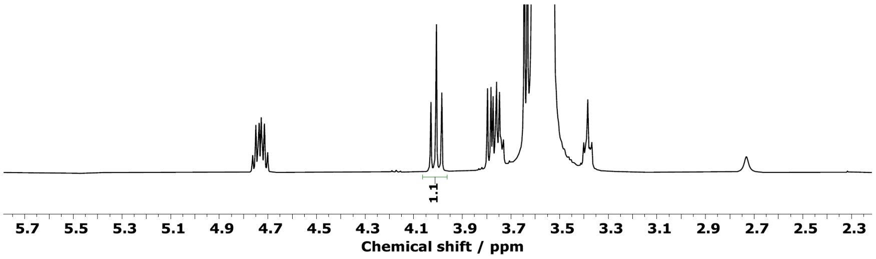

Fig. S8 Product/reagent region of ^{1}H-NMR spectrum of products **7a**/**7b** from GC/ FC_6H_4NCO coupling in THF with LiBr.

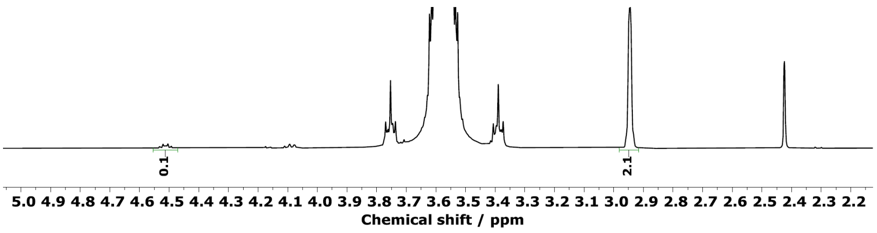

Fig. S9 Product/reagent region of ^{1}H-NMR spectrum of products **8a**/**8b** from CHO/ FC_6H_4NCO coupling in THF with LiBr.

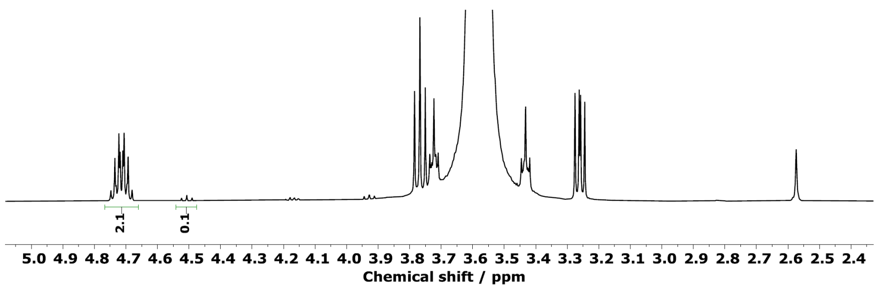

Fig. S10 Product/reagent region of ^{1}H-NMR spectrum of products **9a/9b** from PO/ $Me_2C_6H_3NCO$ coupling in THF with LiBr.

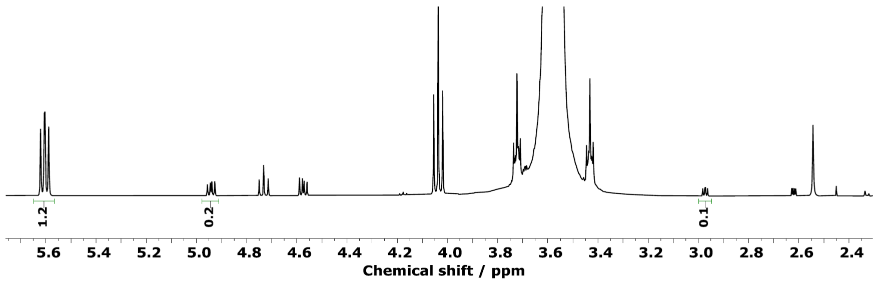

Fig. S11 Product/reagent region of ^{1}H-NMR spectrum of products **10a/10b** from SO/ $Me_2C_6H_3NCO$ coupling in THF with LiBr.

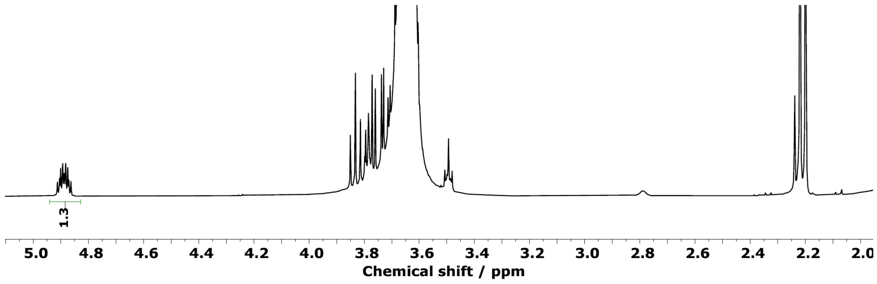

Fig. S12 Product/reagent region of ^{1}H-NMR spectrum of products **11a/11b** from GC/ $Me_2C_6H_3NCO$ coupling in THF with LiBr.

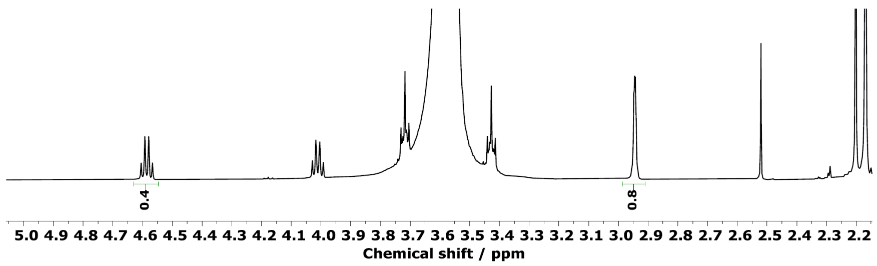

Fig. S13 Product/reagent region of ^{1}H-NMR spectrum of products **12a/12b** from CHO/ $Me_2C_6H_3NCO$ coupling in THF with LiBr.

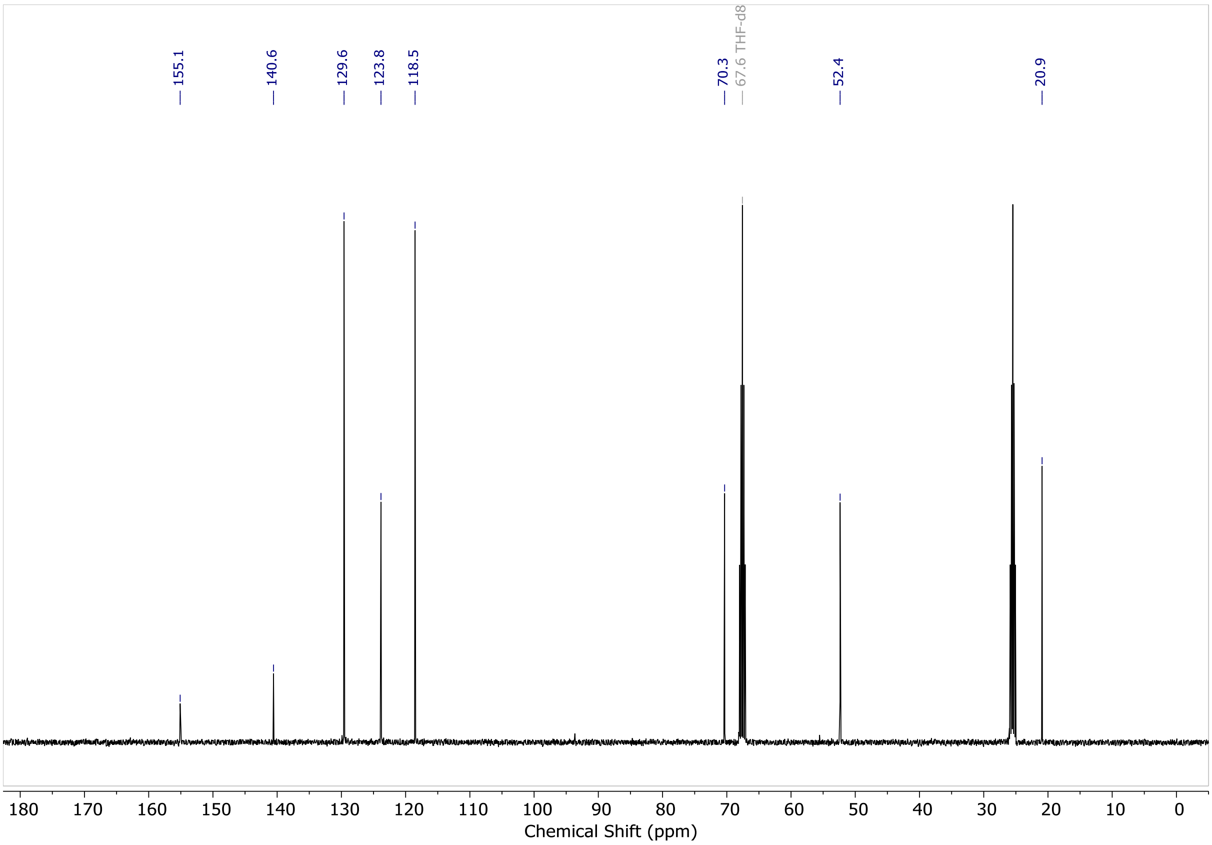

Fig. S14 ^{13}C-NMR spectrum of *5-methyl-3-phenyloxazolidin-2-one* (**1a**) in THF-d_8. The pentet at 25.5 ppm corresponds to THF in this and following spectra.

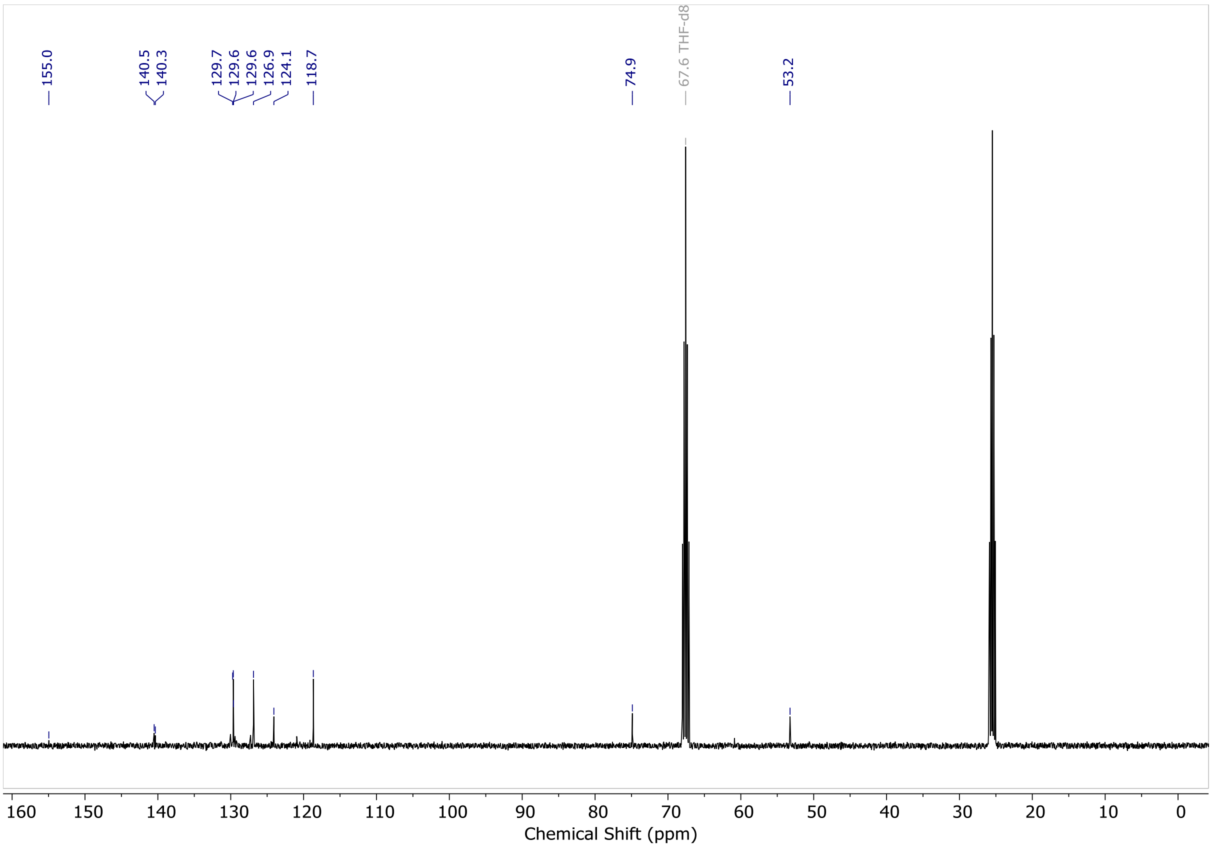

Fig. S15 ^{13}C-NMR spectrum of *3,5-diphenyloxazolidin-2-one* (**2a**) in THF-d_8

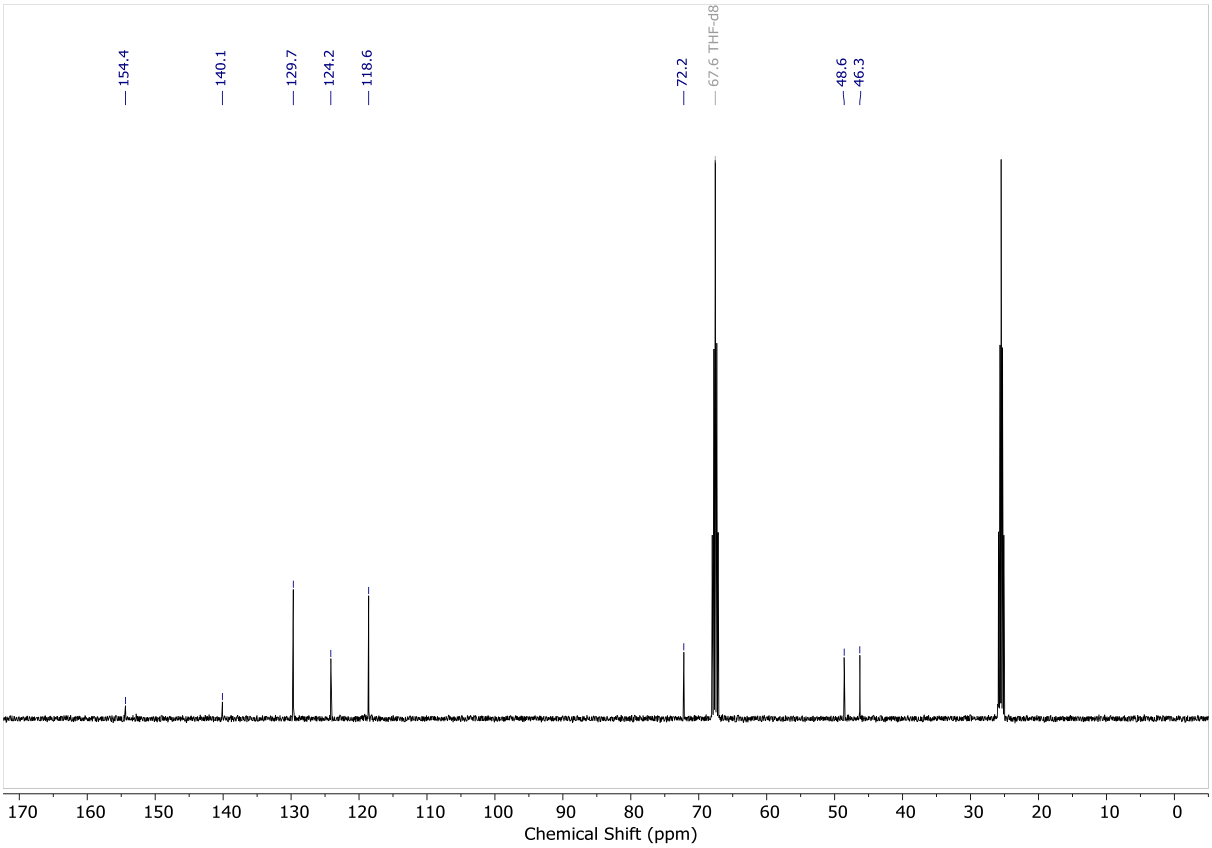

Fig. S16 ^{13}C-NMR spectrum of *5-(chloromethyl)-3-phenyloxazolidin-2-one* (**3a**) in THF-d_8

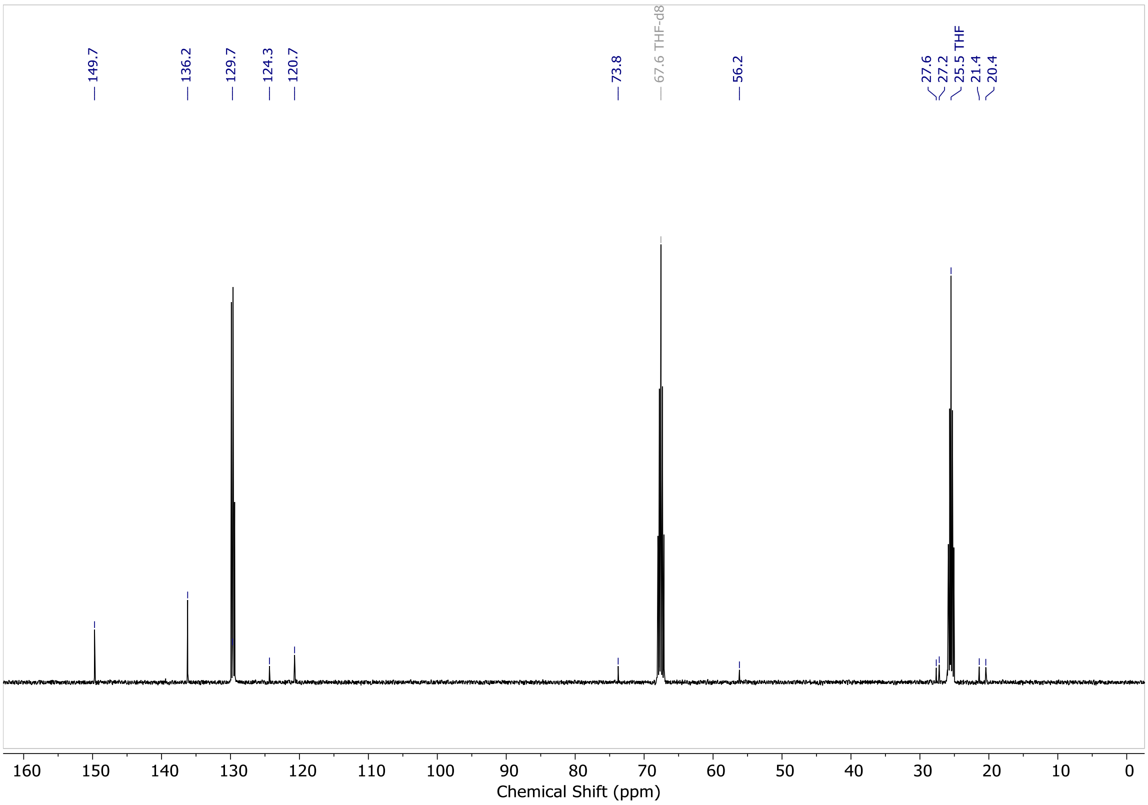

Fig. S17 ^{13}C-NMR spectrum of *4,5-tetramethylene-3-phenyl-1,3-oxazolidin-2-one* (**4a**) in THF-d_8. Peaks at ≈130 ppm may be a phenyisocyanate side-product, evidenced by the overall low conversion.

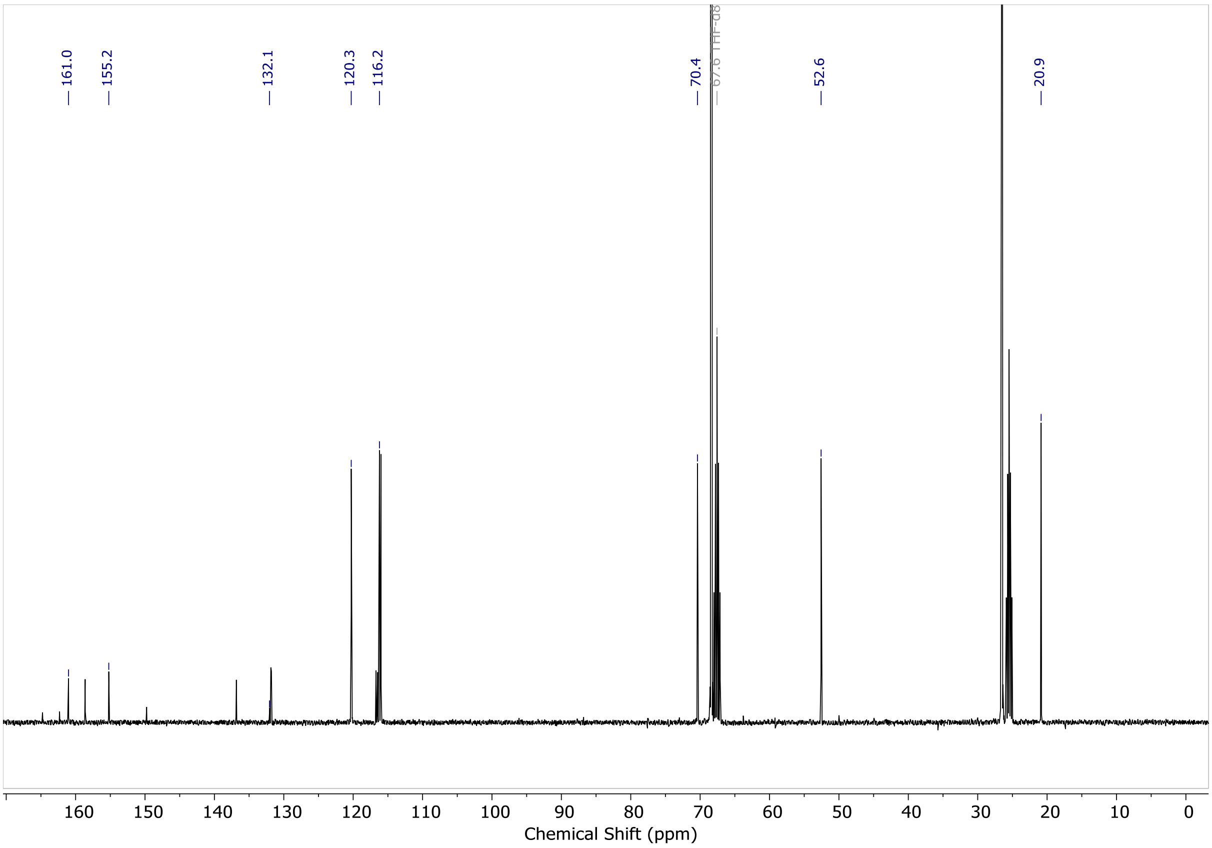

Fig. S18 ^{13}C-NMR spectrum of *3-(4-fluorophenyl)-5-methyloxazolidin-2-one* (**5a**) in THF-d_8. Singlet peaks at 26.5 and 68.4 ppm correspond to isopropyl alcohol in this and following spectra.

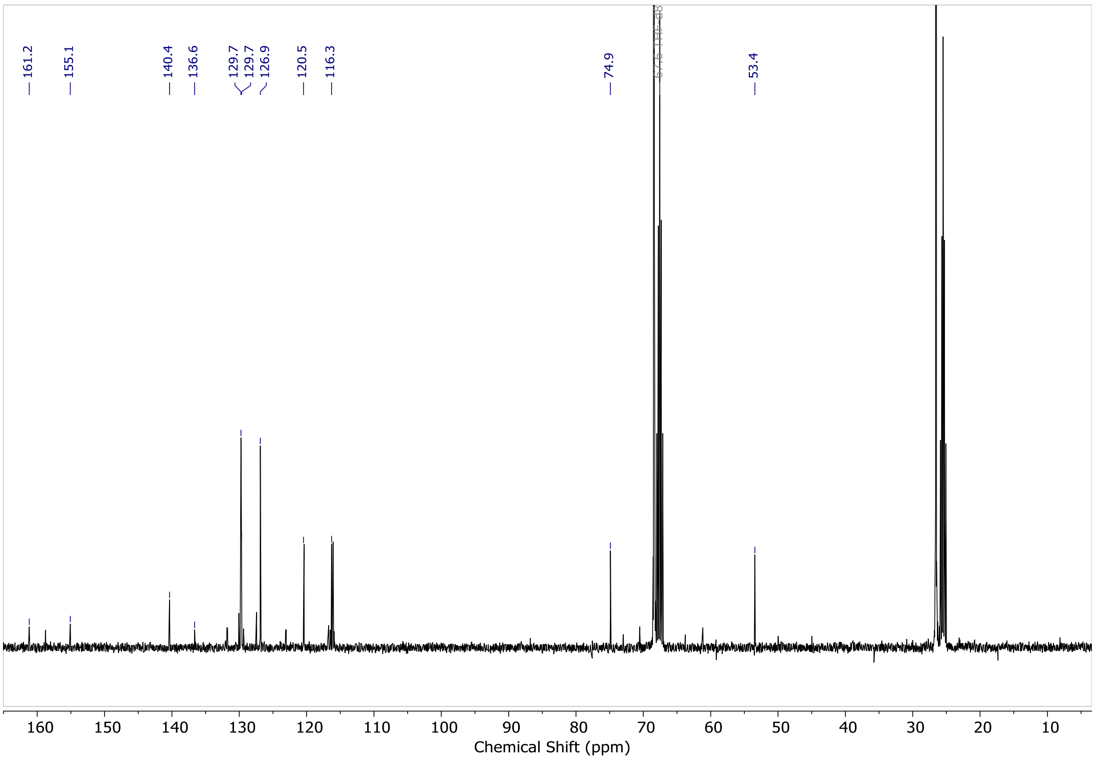

Fig. S19 ^{13}C-NMR spectrum of *3-(4-fluorophenyl)-5-phenyloxazolidin-2-one* (**6a**) in THF-d_8

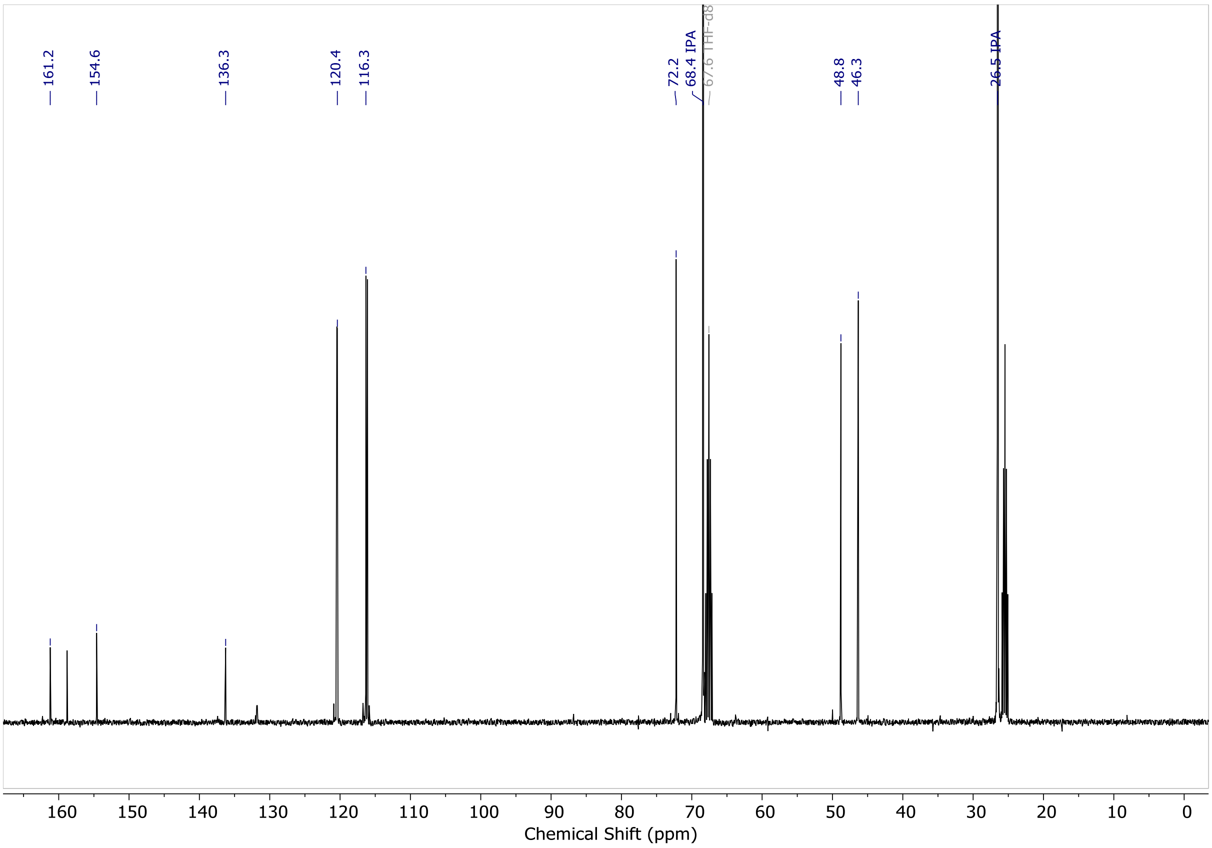

Fig. S20 ^{13}C-NMR spectrum of *3-(4-fluorophenyl)-5-chloromethyloxazolidin-2-one* (**7a**) in THF-d_8

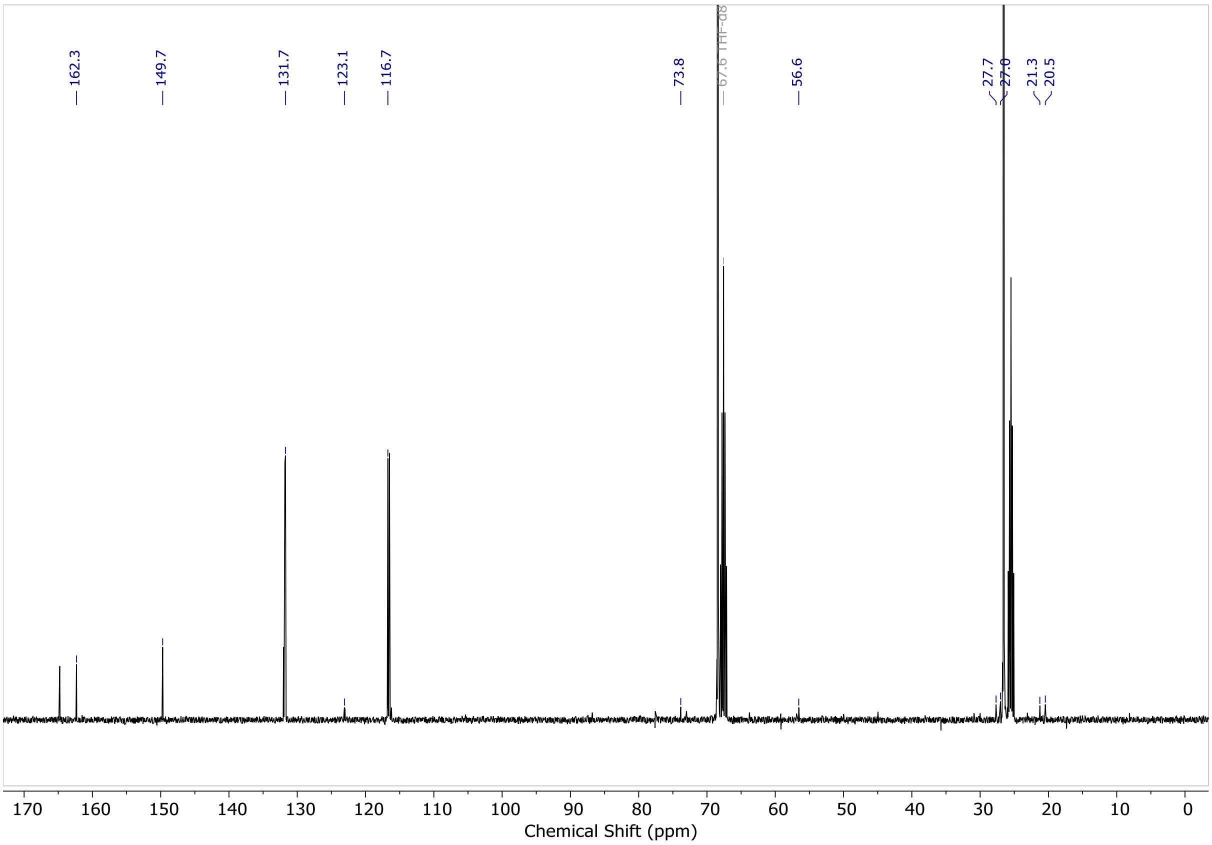

Fig. S21 ^{13}C-NMR spectrum of *3-(4-fluorophenyl)-hexahydrobenzooxazol-2-one* (**8a**) in THF-d_8

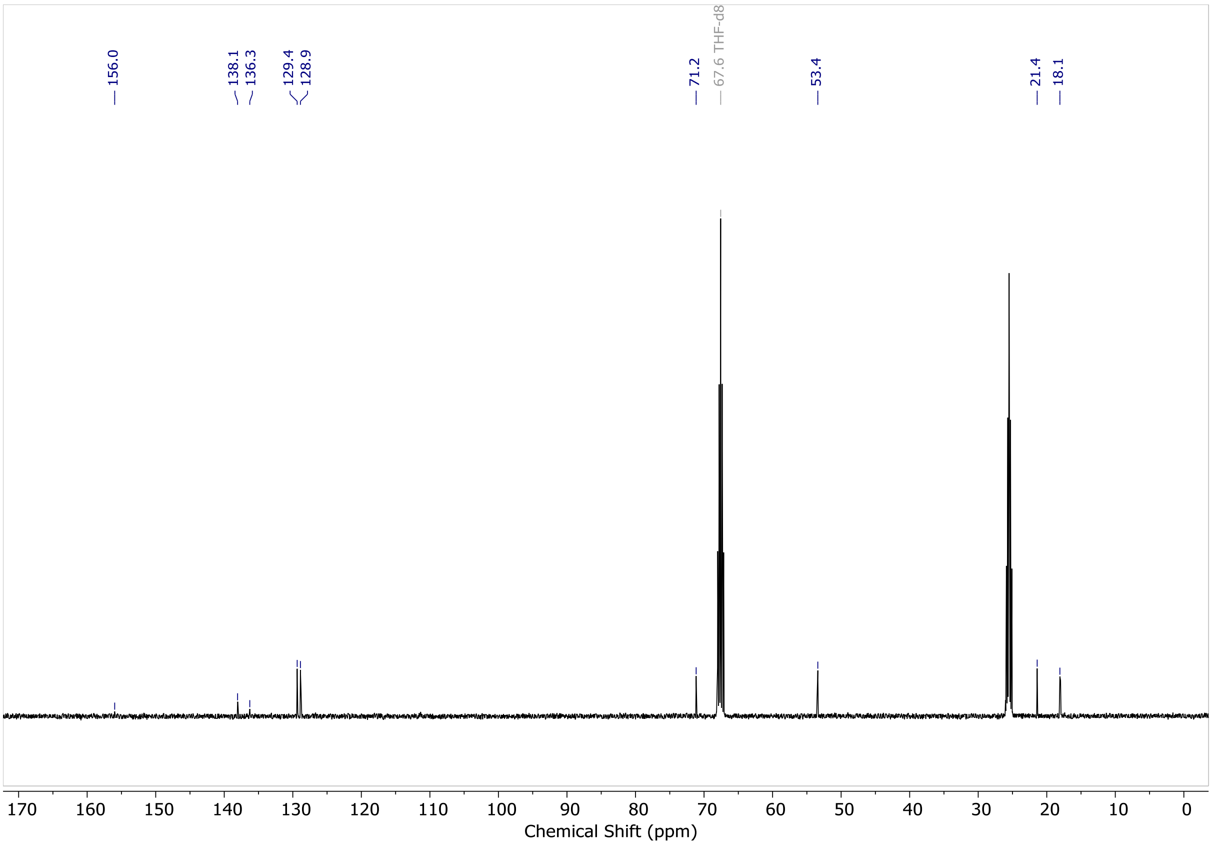

Fig. S22 ^{13}C-NMR spectrum of *3-(2,6-dimethylphenyl)-5-methyloxazolidin-2-one* (**9a**) in THF-d_8

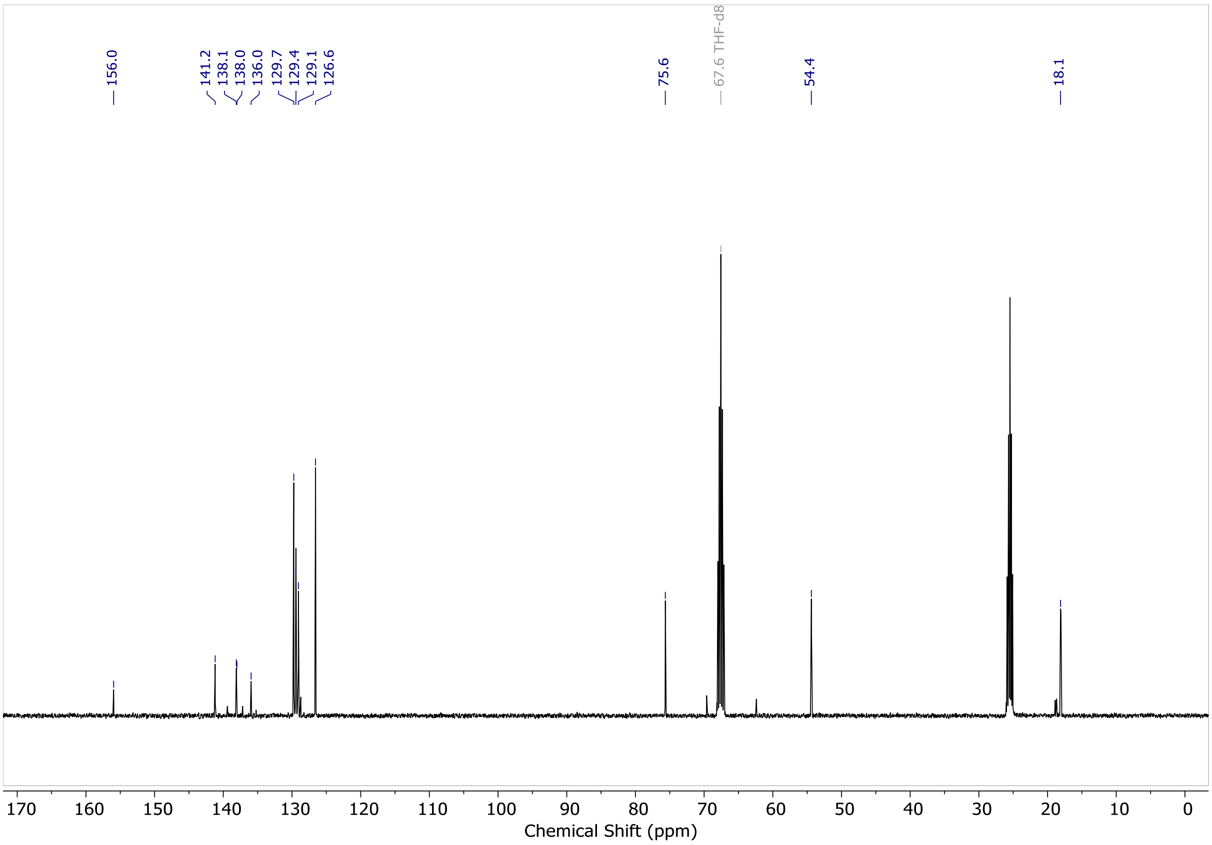

Fig. S23 ^{13}C-NMR spectrum of *3-(2,6-dimethylphenyl)-5-phenyloxazolidin-2-one* (**10a**) in THF-d_8

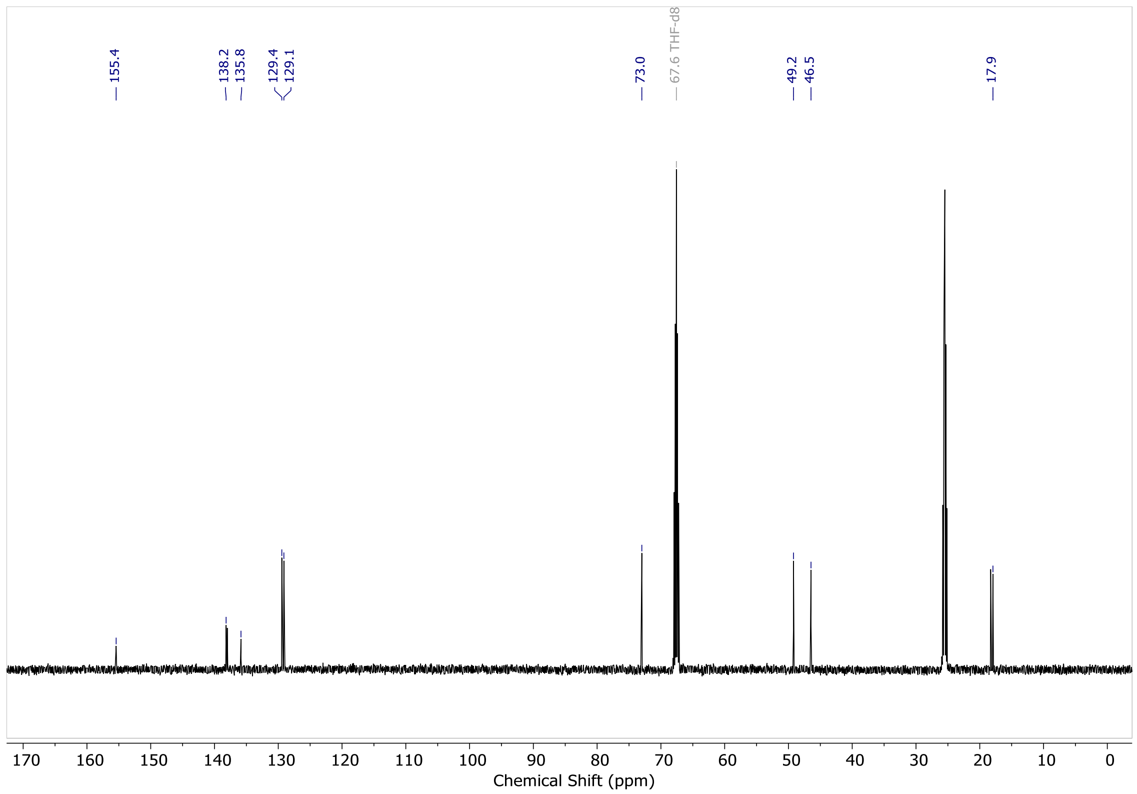

Fig. S24 ^{13}C-NMR spectrum of *5-(chloromethyl)-3-(2,6-dimethylphenyl)oxazolidin-2-one* (**11a**) in THF-d$_8$

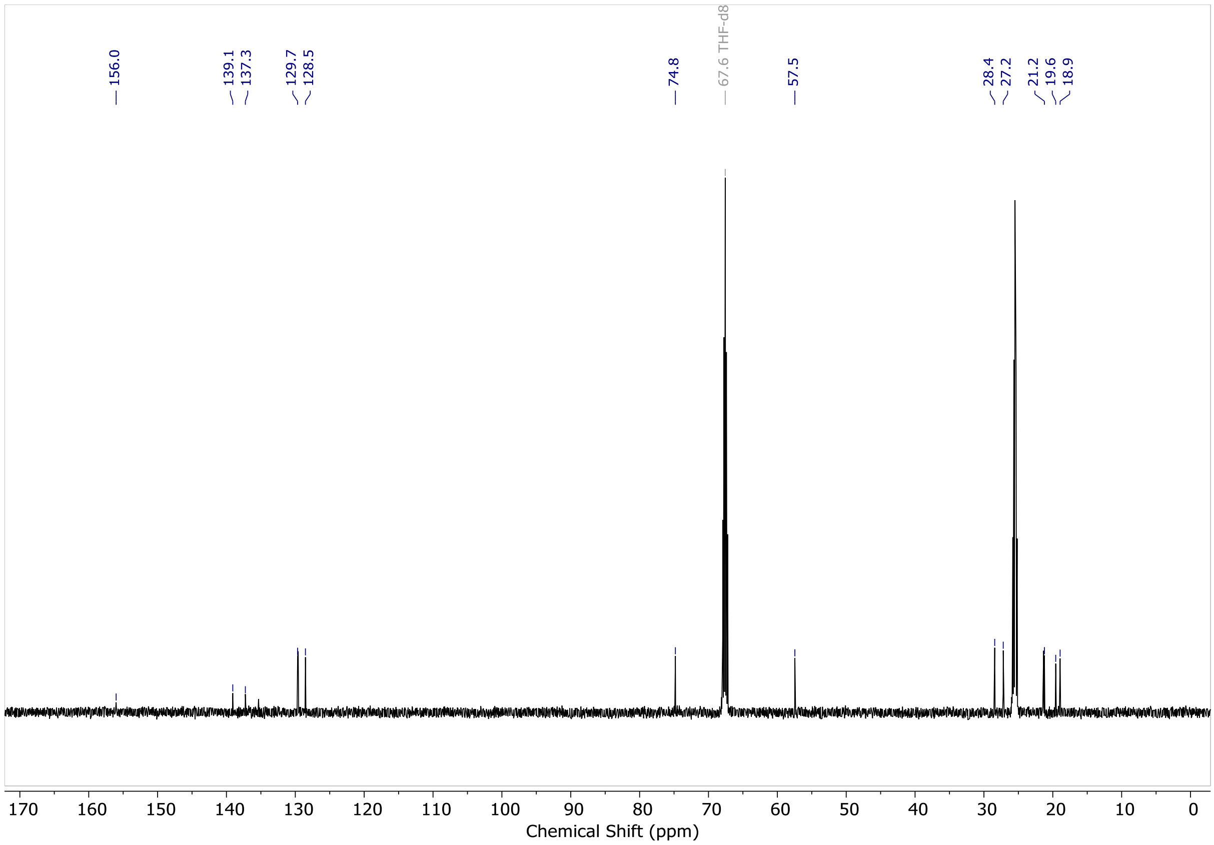

Fig. S25 ^{13}C-NMR spectrum of *3-(2,6-dimethylphenyl)hexahydrobenzo[d]oxazol-2(3H)-one* (**12a**) in THF-d$_8$

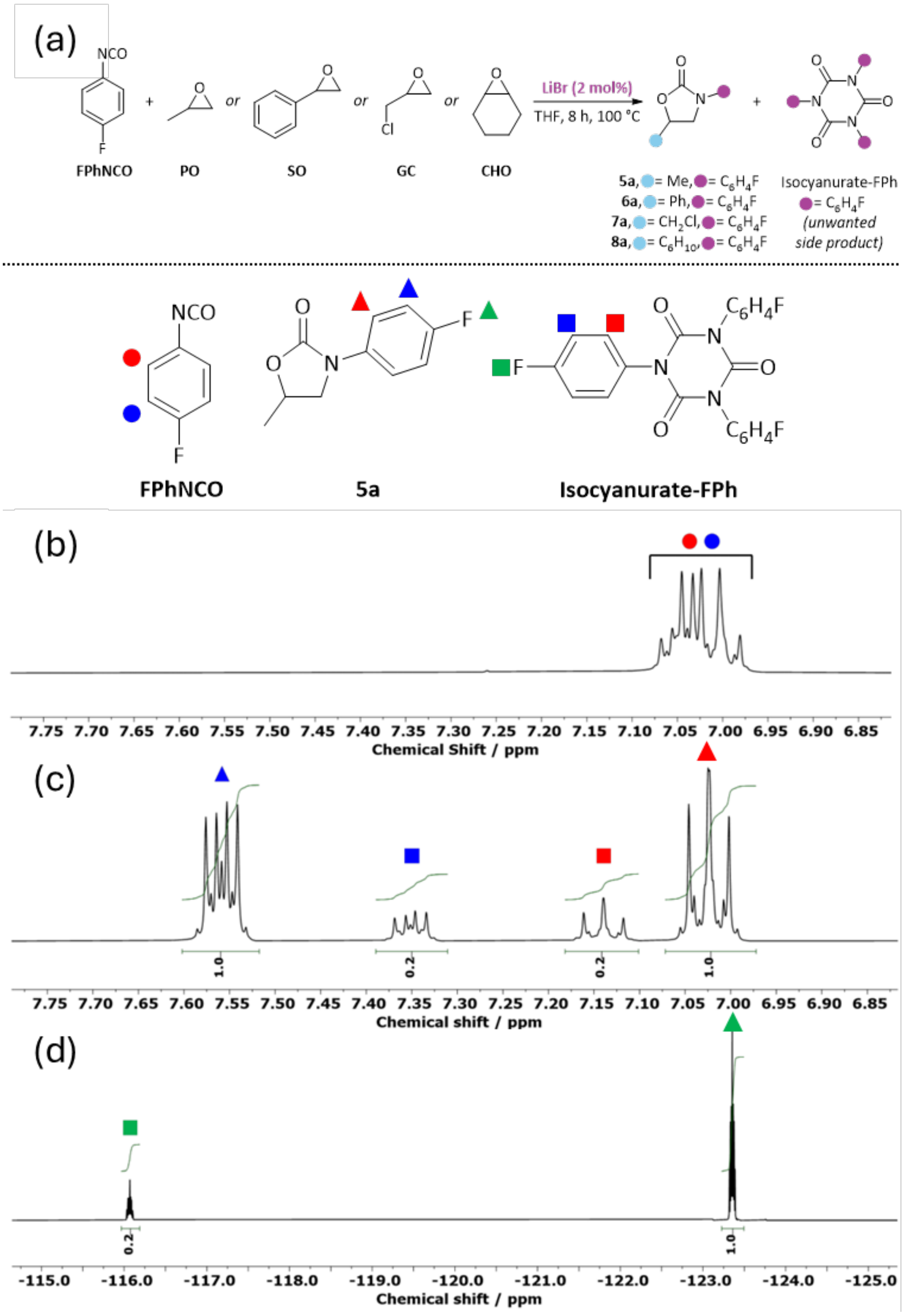

Fig. S26 (a) Isocyanurate side product generated from 1-fluoro-4-isocyanatobenzene (FC_6H_4NCO) with either propylene oxide (PO), styrene oxide (SO), glycidyl chloride (GC) or cyclohexene oxide (CHO). (b) ^{1}H-NMR spectrum of the aromatic region of FC_6H_4NCO from **5a** formation (c) ^{1}H-NMR spectrum of post-precipitation crude product from **5a** formation. (d) ^{19}F-NMR spectrum of post-precipitation crude product from **5a** formation. All spectra shown taken at 400 MHz, $CDCl_3$, 295 K.

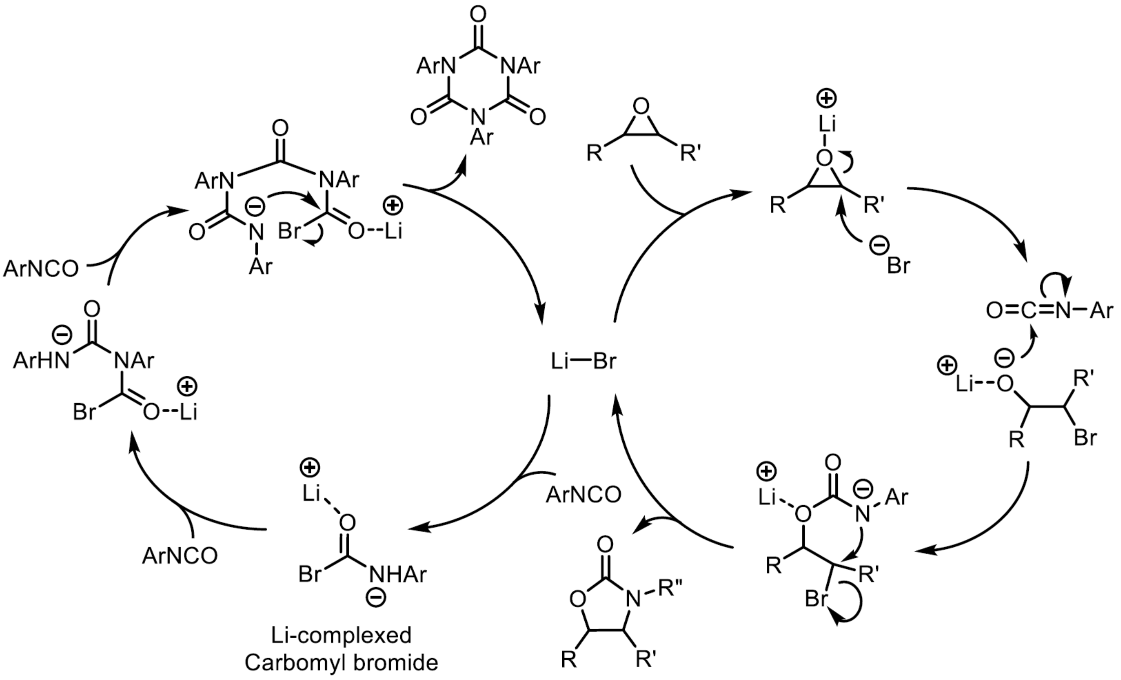

R' = H gives 3,5-isomer **a**
R = H gives 3,4-isomer **b**

Fig. S27 Proposed catalytic cycles for the formation of cyclic carbamates and isocyanurates meditated by LiBr. The 3-5 and 3-4 regioisomers of any cyclic carbamates are named as **a** and **b** respectively.

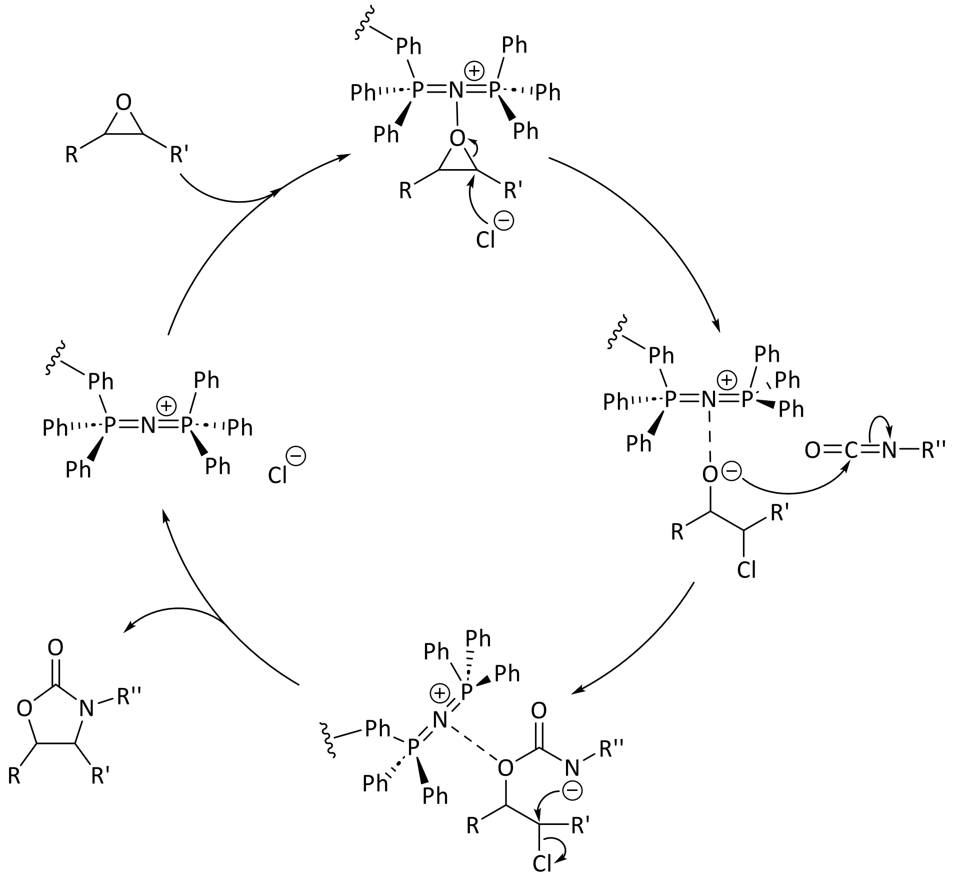

Fig. S28 Proposed catalytic cycle for the formation of cyclic carbamates and isocyanurates meditated by poly(PPNCl). Trimerization does not occur, theorised to be because of the PPNCl-cation steric bulk.

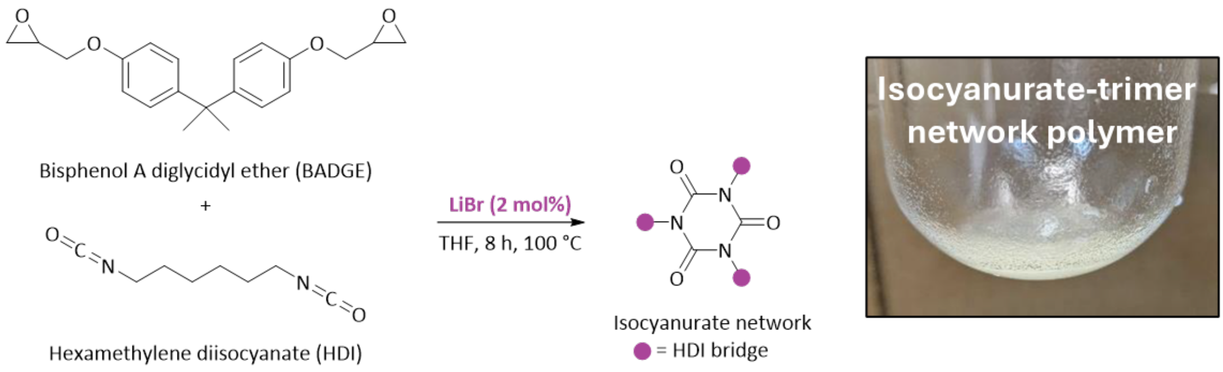

Fig. S29 Reaction of BADGE with HDI leading to formation of a cross-linked isocyanurate polymer, with no cyclic carbamate formation observed.

Conversions, yields and ^{1}H-NMR, ^{13}C-NMR, ^{19}F-NMR and FTIR characterisations of compounds **1a–12a**

5-methyl-3-phenyloxazolidin-2-one (**1a**)

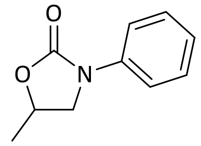

1a

Conversion = >99%, **Yield** = 0.23 g, 72%

^{1}H-NMR (400 MHz, 295 K, THF-d_8) (δ / ppm): 7.52 – 7.45 (m, 2H, ArH), 7.22 – 7.15 (m, 2H, ArH), 6.92 (tt, J = 7.0, 1.0 Hz, 1H, ArH), 4.60 (ddq, J = 8.0, 7.0, 6.0 Hz, 1H, O-CH-CH3), 3.99 (t, J = 8.5 Hz, 1H, N-CHH), 3.55 – 3.40 (dd, J = 8.5, 7.0 Hz, 2H, N-CHH), 1.33 (d, J = 6.0 Hz, 3H, CH-CH3)

^{13}C-NMR (126 MHz, 295 K, THF-d_8) (δ / ppm): 155.1, 140.6, 129.6, 123.8, 118.5, 70.3, 52.4, 20.9

FTIR (cm^{-1}): 1743 (C=O stretch)

3,5-diphenyloxazolidin-2-one (**2a**)

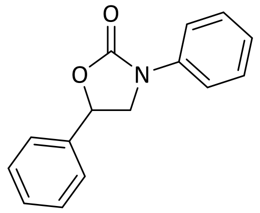

2a

Conversion = 93%, **Yield** = 0.26 g, 61%

^{1}H-NMR (400 MHz, 295 K, THF-d_8) (δ / ppm): 7.67 – 7.59 (m, 2H, Ar*H*), 7.52 – 7.27 (m, 7H, Ar*H*), 7.05 (tt, *J* = 7.0, 1.0 Hz, 1H, Ar*H*), 5.63 (dd, *J* = 9.0, 7.5 Hz, 1H, O-C*H*), 4.42 (t, *J* = 9.0 Hz, 1H, N-C*H*H), 3.92 (dd, *J* = 9.0, 7.5 Hz, 1H, N-CH*H*)

^{13}C-NMR (126 MHz, 295 K, $CDCl_3$) (δ / ppm): 155.0, 140.5, 140.3, 129.7, 129.6, 129.6, 126.9, 124.1, 118.7, 74.9, 53.2

FTIR (cm^{-1}): 1743 (C=O stretch)

5-(chloromethyl)-3-phenyloxazolidin-2-one (**3a**)

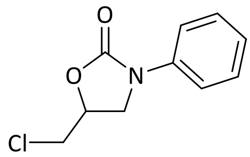

3a

Conversion = >99%, **Yield** = 0.31 g, 82%

^{1}H-NMR (400 MHz, 295 K, THF-d8) (δ / ppm) δ 7.66 – 7.58 (m, 2H, Ar*H*), 7.36 – 7.27 (m, 2H, Ar*H*), 7.10 – 7.01 (m, 1H, Ar*H*), 4.92 – 4.81 (m, 1H, O-C*H*), 4.16 (t, *J* = 9.0 Hz, 1H, N-C*H*H), 3.92 – 3.79 (m, 3H, N-CH*H*, C*H*2-Cl)

^{13}C-NMR (126 MHz, 295 K, THF-d8) (δ / ppm): 154.4, 140.1, 129.7, 124.2, 118.6, 72.2, 48.6, 46.3

FTIR (cm^{-1}): 1734 (C=O stretch)

4,5-tetramethylene-3-phenyl-1,3-oxazolidin-2-one (**4a**)

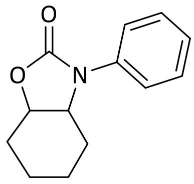

4a

Conversion = 15%, **Yield** = 0.023 g, 5.8%

^{1}H-NMR (400 MHz, 295 K, THF-d_8) (δ / ppm): 7.60 – 7.55 (m, 2H, Ar*H*), 7.33 – 7.26 (m, 2H, Ar*H*), 7.04 (tt, *J* = 7.0, 1.0 Hz, 1H, Ar*H*), 4.56 (dt, *J* = 6.5, 4.0 Hz, 1H, O-C*H*), 4.34 (dt, *J* = 8.5, 6.0 Hz, 1H, N-C*H*), 1.64 – 1.20 (m, 6H, alkyl)

^{13}C-NMR (126 MHz, 295 K, THF-d_8) (δ / ppm): 149.7, 136.2, 129.7, 124.3, 120.7, 73.8, 56.2, 27.6, 27.2, 21.4, 20.4

FTIR (cm^{-1}): 1727 (C=O stretch)

3-(4-fluorophenyl)-5-methyloxazolidin-2-one (**5a**)

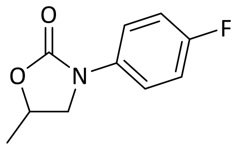

5a

Conversion = 84%

^{1}H-NMR (400 MHz, 295 K, THF-d_8) (δ / ppm): 7.61 – 7.50 (m, 2H, Ar*H*), 7.17 – 7.11 (m, 2H, Ar*H*), 4.67 (ddd, *J* = 8.0, 7.0, 6.0 Hz, 1H, O-C*H*), 4.05 (t, *J* = 8.0 Hz, 2H, N-C*H*H), 3.58 (dd, *J* = 7.0 Hz, 1H, N-CH*H*), 1.40 (d, *J* = 6.0 Hz, 3H, CH-C*H*3)

^{13}C-NMR (126 MHz, 295 K, THF-d_8) (δ / ppm): 161.0 (d, $^1J_{CF}$ = 241.6 Hz), 155.2, 132.1(d, $^4J_{CF}$ = 3.3 Hz), 120.3 (d, $^3J_{CF}$ = 7.8 Hz), 116.2 (d, $^2J_{CF}$ = 22.4 Hz), 70.4, 52.6, 20.9

^{19}F-NMR (376 MHz, 298 K, THF-d_8) (δ / ppm): 123.4

FTIR (cm^{-1}): 1742 (C=O stretch)

3-(4-fluorophenyl)-5-phenyloxazolidin-2-one (**6a**)

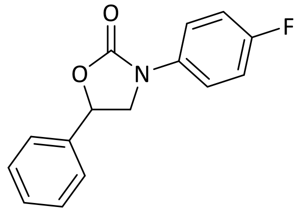

6a

Conversion = 82%

^{1}H-NMR (400 MHz, 295 K, THF-d_8) (δ / ppm): 7.71 – 7.52 (m, 2H, Ar*H*), 7.08 – 7.02 (m, 2H, Ar*H*), 5.60 (dd, *J* = 7.5 Hz, 1H, O-C*H*), 4.37 (t, *J* = 9.0 Hz, 1H, N-C*H*H), 3.88 (dd, *J* = 9.0, 7.5 Hz, 1H, N-CH*H*)

^{13}C-NMR (126 MHz, 295 K, THF-d_8) (δ / ppm): 161.2 (d, $^1J_{CF}$ = 241.6 Hz), 155.1, 140.4, 136.6 (d, $^4J_{CF}$ = 3.3 Hz),129.7, 129.7, 126.9, 120.5 (d, $^3J_{CF}$ = 7.8 Hz), 116.3 (d, $^2J_{CF}$ = 22.4 Hz), 74.9, 53.4

^{19}F-NMR (376 MHz, 298 K, THF-d_8) (δ / ppm): 123.1

FTIR (cm^{-1}): 1746 (C=O stretch)

3-(4-fluorophenyl)-5-chloromethyloxazolidin-2-one (**7a**)

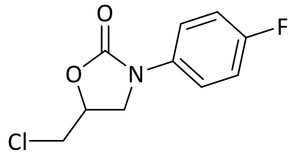

7a

Conversion = 89%

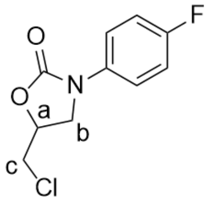

^{1}H-NMR (400 MHz, 295 K, THF-d_8) (δ / ppm): 7.63 – 7.47 (m, 2H, Ar*H*), 7.02 (dd, *J* = 9.0, 8.0 Hz, 2H, Ar*H*), 4.82 (a, ddt, *J* = 9.0, 6.0, 5.0 Hz, 1H, O-C*H*), 4.10 – 3.68 (b & c, m, 4H, N-CH*H*, C*H*2-Cl)

^{13}C-NMR (126 MHz, 295 K, $CDCl_3$) (δ / ppm): 161.2 (d, $^1J_{CF}$ = 241.6 Hz), 154.6, 136.3 (d, $^4J_{CF}$ = 3.3 Hz), 120.4 (d, $^3J_{CF}$ = 7.8 Hz), 116.3 (d, $^2J_{CF}$ = 22.4 Hz), 72.2 (a), 48.8 (b), 46.3 (c)

^{19}F-NMR (376 MHz, 298 K, $CDCl_3$) (δ / ppm): 118.7

FTIR (cm^{-1}): 1731 (C=O stretch)

3-(4-fluorophenyl)-hexahydrobenzooxazol-2-one (**8a**)

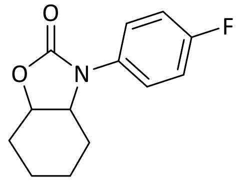

8a

Conversion = 4.8%

^{1}H-NMR (400 MHz, 295 K, THF-d_8) (δ / ppm): 7.40 – 7.30 (m, 2H, Ar*H*), 7.00 – 6.91 (m, 2H, Ar*H*), 4.56 (dt, *J* = 6.5, 4.5 Hz, 1H, O-C*H*), 4.17 – 4.09 (m, 1H, N-C*H*), 2.07 – 2.03 (m, 1H, CH-C*H*2), 1.94 – 1.90 (m, 1H, CH-C*H*2), 1.53 – 1.20 (m, 4H, CH2-C*H*2-C*H*2-CH2)

^{13}C-NMR (126 MHz, 295 K, THF-d_8) (δ / ppm): 162.3 (d, $^1J_{CF}$ = 241.6 Hz), 149.7, 132.0 (d, $^4J_{CF}$ = 3.3 Hz), 123.1 (d, $^3J_{CF}$ = 7.8 Hz), 116.4 (d, $^2J_{CF}$ = 22.4 Hz), 73.8, 56.6, 27.7, 27.0, 21.3, 20.5

^{19}F-NMR (376 MHz, 298 K, THF-d_8) (δ / ppm): 116.1

FTIR (cm^{-1}): 1744 (C=O stretch)

3-(2,6-dimethylphenyl)-5-methyloxazolidin-2-one (**9a**)

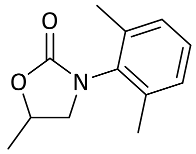

9a

Conversion = 97%, **Yield** = 0.32 g, 87%

^{1}H-NMR (400 MHz, 295 K, THF-d_8) (δ / ppm): 7.18 – 6.97 (m, 3H, Ar*H*), 4.77 (dd, *J* = 8.0, 6.5 Hz, 1H, O-C*H*), 3.87 (t, *J* = 8.5 Hz, 1H, N-C*H*H), 3.36 (dd, *J* = 8.5, 6.5 Hz, 1H, N-CH*H*), 2.24 (s, 6H, Ar-C*H*3), 1.47 (d, *J* = 6.0 Hz, 3H, CH-C*H*3)

^{13}C-NMR (126 MHz, 295 K, THF-d_8) (δ / ppm): 156.0, 138.1, 136.3, 129.4, 128.9, 71.2, 53.4, 21.4, 18.1

FTIR (cm^{-1}): 1743 (C=O stretch)

3-(2,6-dimethylphenyl)-5-phenyloxazolidin-2-one (**10a**)

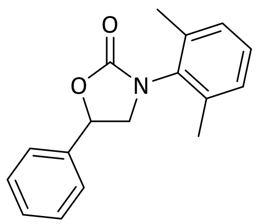

10a

Conversion = 94%, **Yield** = 0.38 g, 79%

^{1}H-NMR (400 MHz, 295 K, THF-d_8) (δ / ppm): 7.51 – 7.45 (m, 2H, Ar*H*), 7.45 – 7.38 (m, 2H, Ar*H*), 7.37 – 7.32 (m, 2H, Ar*H*), 7.14 – 7.01 (m, 2H, Ar*H*), 5.73 (dd, *J* = 9.0, 7.0 Hz, 1H, O-C*H*), 4.17 (t, *J* = 9.0 Hz, 1H, N-C*H*H), 3.63 (dd, *J* = 9.0, 7.0 Hz, 1H, N-CH*H*), 2.32 (s, 3H, Ar-C*H*3), 2.15 (s, 3H, Ar-C*H*3)

^{13}C-NMR (126 MHz, 295 K, $CDCl_3$) (δ / ppm): 156.0, 141.2, 138.1, 138.0, 136.0, 129.7, 129.4, 129.1, 126.6, 75.6, 54.4, 18.1

FTIR (cm^{-1}): 1739 (C=O stretch)

5-(chloromethyl)-3-(2,6-dimethylphenyl)oxazolidin-2-one (**11a**)

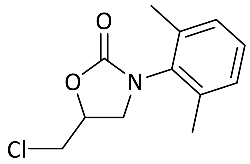

11a

Conversion = >99%, **Yield** = 0.36 g, 83%

^{1}H-NMR (400 MHz, 295 K, THF-d_8) (δ / ppm): 7.14 – 7.05 (m, 3H, Ar*H*), 4.94 (ddt, *J* = 9.0, 6.0, 4.5 Hz, 1H, O-C*H*), 3.93 – 3.82 (m, 3H, N-C*H*H, C*H*2-Cl), 3.67 (dd, *J* = 9.0, 6.0 Hz, 1H, N-CH*H*), 2.26 (s, 3H, Ar-C*H*3), 2.25 (s, 3H, Ar-C*H*3)

^{13}C-NMR (126 MHz, 295 K, THF-d_8) (δ / ppm): 155.4, 138.2, 135.8, 129.4, 129.1, 73.0, 49.2, 46.5, 17.9

FTIR (cm^{-1}): 1747 (C=O stretch)

3-(2,6-dimethylphenyl)hexahydrobenzo[d]oxazol-2(3H)-one (**12a**)

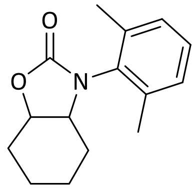

12a

Conversion = 31%, **Yield** = 0.097 g, 22%

^{1}H-NMR (400 MHz, 295 K, THF-d_8) (δ / ppm): 7.12 – 7.03 (m, 3H, Ar*H*), 4.67 (q, *J* = 6.0 Hz, 1H, O-C*H*), 4.15 (q, *J* = 6.0 Hz, 1H, N-C*H*), 2.31 (s, 3H, Ar-C*H*3), 2.23 (s, 3H, Ar-C*H*3), 1.92 (dtt, *J* = 7.0, 5.0, 2.5 Hz, 2H, CH-C*H*2), 1.70 – 1.23 (m, 6H, CH-C*H*2, CH2-C*H*2-C*H*2-CH2)

^{13}C-NMR (126 MHz, 295 K, THF-d_8) (δ / ppm): 156.0, 139.1, 137.3, 129.7, 128.5, 74.8, 57.5, 28.4, 27.2, 21.2, 19.6, 18.9

FTIR (cm^{-1}): 1747 (C=O stretch)

References

(1) Wang, M.; Shanmugam, M.; McInnes, E. J.; Shaver, M. P. Light-Induced Polymeric Frustrated Radical Pairs as Building Blocks for Materials and Photocatalysts. *J. Am. Chem. Soc.* **2023**, *145*, 24294-24301.
(2) Xu, Z.; Wang, M.; Shaver, M. P. Polymeric Bis (Triphenylphosphine) Iminium Chloride as a Recyclable Catalyst. *Chem. Sci.* **2024**, *15*, 15745-15750.
